# El Niño-driven phase shift to algal dominance on Isla del Caño’s coral reefs: implications for urgent restoration

**DOI:** 10.7717/peerj.20088

**Published:** 2025-11-20

**Authors:** Caroline V. Palmer, Shanttal Valeria Rodríguez Esquivel, Christopher M. Parker

**Affiliations:** 1 Innoceana, Uvita, Puntarenas, Costa Rica; 2 School of Biological and Marine Sciences, University of Plymouth, Plymouth, Devon, United Kingdom

**Keywords:** Ecological Recovery Feasibility Index, Coral reef degradation, Turf algal dominance, El Niño 2023–24, Coral bleaching, Eastern Tropical Pacific, Costa Rica

## Abstract

**Background:**

The 2023–24 El Niño event caused extreme marine heat stress and widespread coral bleaching. Coral reefs at the Reserva Biológica Isla del Caño and the northern coast of the Osa Peninsula, Costa Rica, underpin critical ecosystem services, including biodiversity conservation and marine tourism, and have previously withstood similar events with minimal coral loss. Evaluating the ecological impacts of the 2023–24 El Niño is essential to assess coral reef resilience and guide future management.

**Methods:**

Coral Reef Watch sea surface temperature (SST) data (1985–2025; CoralTemp V3.1) were used to calculate long-term SST trends and degree heating weeks (DHW). Reef surveys were conducted at nine sites between 2019 and 2025, with primary benthic composition and coral health data collected in 2024–25. Benthic cover was assessed using point-intercept, line-intercept, and quadrat methods, while coral diversity, abundance, and health were measured *via* belt transects. Beta regression was used to assess the effect of temperature on coral cover, and multivariate analyses, including principal component analysis (PCA) and similarity percentage analysis (SIMPER), evaluated benthic community changes and bleaching patterns. An Ecological Recovery Feasibility Index (ERFI) was developed using PCA loadings and benthic indicators to rank sites by recovery potential.

**Results:**

SST increased significantly over the past 40 years (∼0.23 °C/decade), with the 2023–24 El Niño recording peak SST (31.2 °C). Bleaching threshold exceedance days increased, while cool days declined. Twelve coral taxa were recorded; *Pocillopora* spp. and *Porites lobata* were present at all sites. Coral diversity varied, with Cueva and Ancla highest, and San Josecito lowest. Estimated baseline bleaching prevalence was ∼23%, highest in *Pocillopora* spp. (33.9%). SIMPER and PCA revealed a shift from coral to algal dominance: turf algae increased by 70.62%, dead coral declined 80.71%, and coral cover fell 40.44%. Major coral declines were statistically significant at Ancla, Esquina, and Tina. Bayesian regression confirmed coral decline at Chorro, Cueva, Tina, and Ancla, alongside turf algae increases. Coral cover was higher at warmer sites, though non-temperature site-specific factors were also influential. Chorro and Esquina had the highest recovery potential; Ancla, San Josecito, and Barco Profundo the lowest.

**Conclusion:**

There is an urgent need to develop and implement a coral reef restoration strategy for Isla del Caño that addresses site-specific conditions, integrates tourism management, and promotes long-term resilience. Under continued climate change, localized, targeted restoration will be essential to maintain the ecological function of these historically resilient but increasingly vulnerable reefs in Costa Rica’s Eastern Tropical Pacific.

## Introduction

Coral reefs are vital yet sensitive ecosystems that have experienced 50% global loss of coral cover over the last three decades (IPBES et al., 2019). Warm water coral reefs continue to face the unprecedented human-driven threat of global warming, repeatedly exceeding established thermal and bleaching stress thresholds and crossing critical ecological tipping points (Armstrong McKay et al., 2022; Intergovernmental Panel on Climate Change (2022a); Intergovernmental Panel on Climate Change (IPCC), 2022b). Breaching tipping points can result in irreversible shifts from coral to algal dominance (Klein, Roch & Duarte, 2024), reducing biodiversity (Barlow et al., 2018), compromising ecosystem functioning, and threatening livelihoods (Sing Wong, Vrontos & Taylor, 2022). Coral reef collapse typically occurs with 10% coral cover or less, and is associated with low taxa diversity and limited ecological interactions (Armstrong McKay et al., 2022; Darling et al., 2019). Acute events such as El Niño-driven marine heatwaves can cause widespread coral mortality and accelerate ecosystem decline, particularly when reefs are already degraded or near critical thresholds (Pearce-Kelly et al., 2025). Corresponding to the increasing frequency of El Niño Southern Oscillation (ENSO) events (Claar et al., 2018; Heron et al., 2016), there has been a significant increase in the extent of mass coral bleaching over the past 50 years (Virgen-Urcelay & Donner, 2023). The most recent El Niño (2023-24) drove the fourth global mass bleaching event with projected widespread coral mortality (Reimer et al., 2024), underscoring the urgent need for local coral reef monitoring and targeted restoration strategies (Hein et al., 2021; Shaver et al., 2022; Suggett et al., 2024).

Periods of abnormally warm water associated with ENSO have variably driven coral declines in the Eastern Tropical Pacific (ETP), including Isla del Caño (Alvarado et al., 2020; Cortés et al., 2010; Guzmán et al., 1987; Guzmán & Cortés, 2007; Jiménez et al., 2001). Isla del Caño, located in the south Pacific coast of Costa Rica near the Osa Peninsula, was designated a protected area (Biological Reserve) in 1978. The Isla del Caño Biological Reserve, International Union for Conservation of Nature (IUCN) category I Wilderness Area for the long-term protection of ecological integrity (Dudley, 2008), was subsequently expanded to include 55 km^2^ of marine area (Sistema Nacional de Áreas de Conservación (SINAC), 2009). Isla del Caño reportedly harbors the highest coral species richness in continental Costa Rica (Alvarado et al., 2015; Cortés et al., 2010; Salas, Sánchez-Godínez & Montero-Cordero, 2016) and is proposed as a unique biodiversity hotspot that has garnered the dependence of an extensive local dive industry (ACOSA-TNC-UCI-ELAP, 2008; BIOMARCC-SINAC-GIZ, 2016; Naranjo-Arriola, 2021). With their protected status, high ecological and socio-economic importance, the biodiverse reefs of Isla del Caño are key contributors to Costa Rica’s ETP and purported to be resilient to climate change (Friedlander et al., 2022).

The 1982–83 El Niño impacted Isla del Caño, causing 50% coral mortality overall, virtually eliminating populations of *Gardineroseris planulata*, *Porites panamensis*, and *Pocillopora* spp. in shallow reef zones, causing a shift to crustose coralline algae (CCA) dominance in many reef areas (Guzmán et al., 1987). Subsequent El Niño events, however, have had a lesser impact on the coral communities at Isla del Caño. For example, although global sea surface temperatures (SSTs) were reported to be higher in the 1998 coral bleaching event compared to the 1983 event (Wilkinson, 1999), coral mortality was minimal at Isla del Caño at just 5% (Guzmán & Cortés, 2001). However, coral recovery was not observed between the mid 1980s and 1998, with coral cover across all reef habitats reported to be approximately 10% (Guzmán & Cortés, 2001), though increased in subsequent decades. The survival of corals in the 1998 bleaching event, including *Pocillopora* spp., that were impacted in previous thermal events, was suggested to be due to greater tolerance, acclimatization, and marine protected area (MPA) status (Alvarado et al., 2020). However, MPA status offers little protection against warming-associated bleaching and therefore plays a limited role in mitigating climate-change-scale impacts (Johnson, Dick & Pincheira-Donoso, 2022), suggesting that other factors drove apparent local resilience or refugia status of Isla del Caño’s reefs. Similarly, despite the 2015–16 El Niño being a significant global-scale bleaching event (Eakin, Sweatman & Brainard, 2019) that drove losses of 50 to 75% coral cover at Isla del Coco and Golfo Dulce in the Costa Rican ETP, coral mortality was minimal at Isla del Caño, with less than a 4% decrease in coral cover (Alvarado et al., 2020). Consistently, coral cover of 30.3% was reported between 2016 and 2017 (Naranjo-Arriola, 2021). Whilst Friedlander et al. (2022) reported coral cover of coral reef areas to be approximately 20% in 2019, ecological monitoring of Isla del Caño as part of Costa Rica’s National Ecological Monitoring Programme (Programa Nacional de Monitoreo Ecológico; PRONAMEC; Sistema Nacional de Áreas de Conservación (SINAC), 2016) reported coral cover between December 2015 and September 2023 to range from 23.2% to over 70% across five reef sites (Sistema Nacional de Áreas de Conservación (SINAC), 2023). The PRONAMEC protocol defines healthy reefs as having between 30 and 70% coral cover with a reduction in coral cover of ≥11% and a ≥21% increase in algal cover warranting comprehensive management measures to reduce impacts (Sistema Nacional de Áreas de Conservación (SINAC), 2016 p. 23). Additionally, rocky reefs at Isla del Caño, are considered healthy with up to 40% turf algae, and a more than 21% change considered problematic (SINAC-UNA, 2021, p. 39). However, it is unknown whether the reefs of Isla del Caño retained coral cover and their purported resilience during the 2023-24 El Niño.

In response to increasingly frequent and extreme climate events, such as El Niño, and significant coral die-offs, coral reef restoration efforts are accelerating worldwide (Boström-Einarsson et al., 2020; Edwards, Guest & Humanes, 2024; Suggett et al., 2025), with the general aim of improving the ecological state. Whilst coral reef restoration projects have flourished along Costa Rica’s coastlines (Alvarado et al., 2025), they have not, been established at Isla del Caño, with the exception of a small-scale trial in response to the coral loss of the 1982–83 El Niño (Guzmán, 1991). The lack of restoration at Isla del Caño is likely historically due, in part, to a lack of need given the minimal coral declines reported for recent El Niño events (Guzmán et al., 1987; Jiménez et al., 2001) and the resilient status assigned to the coral communities (Alvarado et al., 2020; Friedlander et al., 2022). Given the increasing threat of climate change to coral reef stability (Armstrong McKay et al., 2022; Intergovernmental Panel on Climate Change, 2022a) and the 2023–24 El Niño mass bleaching event (Reimer et al., 2024), there may be an increasing need to consider potential conservation and restoration interventions at Isla del Caño (Shaver et al., 2020; Van Oppen et al., 2017). To determine the necessity and feasibility of restoration efforts or resilience strategies at Isla del Caño, fine-scale information on the impact of warming events is needed (Suggett et al., 2025) coupled with a detailed assessment of the current health status of the reef communities (SINAC-GIZ, 2020). If the need for coral reef restoration intervention is identified a strategic plan for locally targeted interventions can be developed (Kleypas et al., 2021; Shaver et al., 2022; SINAC-GIZ, 2020; Suggett et al., 2024), in line with local technical and ethical guidelines (Alvarado et al., 2025; SINAC-GIZ, 2020).

In other areas of restoration ecology, composite indices are increasingly used to integrate ecological and environmental data into interpretable, site-level metrics that guide prioritization and planning (Orsi, Geneletti & Newton, 2011; Riedler et al., 2015; Smith et al., 2023b). These tools synthesize diverse indicators, such as species composition, ecological distance from reference communities, or habitat quality, into a single measure to inform targeted restoration (Orsi, Geneletti & Newton, 2011). The coral reef restoration community has similarly called for structured, science-based frameworks to guide interventions, especially in data-limited contexts (Shaver et al., 2020). With sufficient baseline information, such indices offer an opportunity to identify reef sites with limited natural recovery potential and to triage restoration resources effectively.

The aims of this study were to assess the ecological impacts of the 2023–24 El Niño event on coral reefs at Isla del Caño and the northern Osa Peninsula, Costa Rica, and to explore the potential need for management interventions. To contextualize the thermal event, we analyzed four decades of satellite-derived SST data. Shifts in benthic composition across nine sites were explored, with a focus on coral loss and algal proliferation. To explore the need for restoration and to inform site-specific management, a composite, multi-metric index of the potential for natural reef recovery was developed.

## Materials & Methods

### Sea surface temperature

Daily SST data for Isla del Caño (8°42′59″N 83°53′06″W) were obtained from Coral Reef Watch 5 km Heat Stress Product (1985–2025; CoralTemp V3.1; Heron et al., 2015; Skirving et al., 2020). The Maximum of Monthly Means of climatological SST (MMM) was computed for 1985–1990 and 1993, excluding 1991–1992 due to anomalies caused by the Mt. Pinatubo eruption (Heron et al., 2016). Degree Heating Weeks (DHW), indicative of thermal stress accumulation, were calculated as days exceeding MMM + 0.5 °C, using a 12-week rolling sum and dividing this by seven to express thermal stress in °C-weeks (Liu, Strong & Skirving, 2003; Liu et al., 2018). MMM + 0.5 °C was used instead of MMM + 1 °C to increase the sensitivity for capturing localized bleaching events (Lachs et al., 2023). The bleaching threshold was defined as MMM + 0.5 °C and the cooling threshold was defined as the climatological monthly mean (1985–1990 and 1993) minus 0.5 °C. This value represents a lower thermal bound used to identify days that may contribute to cold-water exposure or thermal relief, potentially moderating heat stress impacts. Changes in the frequency of days breaching this cooling threshold were analyzed to assess whether such thermal buffering has declined over time. A linear regression model was used to assess the long-term SST trends, with date as the predictor, and the slope estimate was used to calculate annual and decadal warming. The influence of ENSO on SST variability was also explored using linear regression, using date and Oceanic Niño Index (ONI) as the predictor variables. Diagnostic residual *vs.* time and normal Q-Q plots indicated that heteroscedasticity and outliers had minimal impact on the model. The change in annual frequency of days breaching bleaching and cooling thresholds between 1985 and 2025 were explored using linear regression models.

### Study area, survey methods and environmental parameters

**Figure 1:**
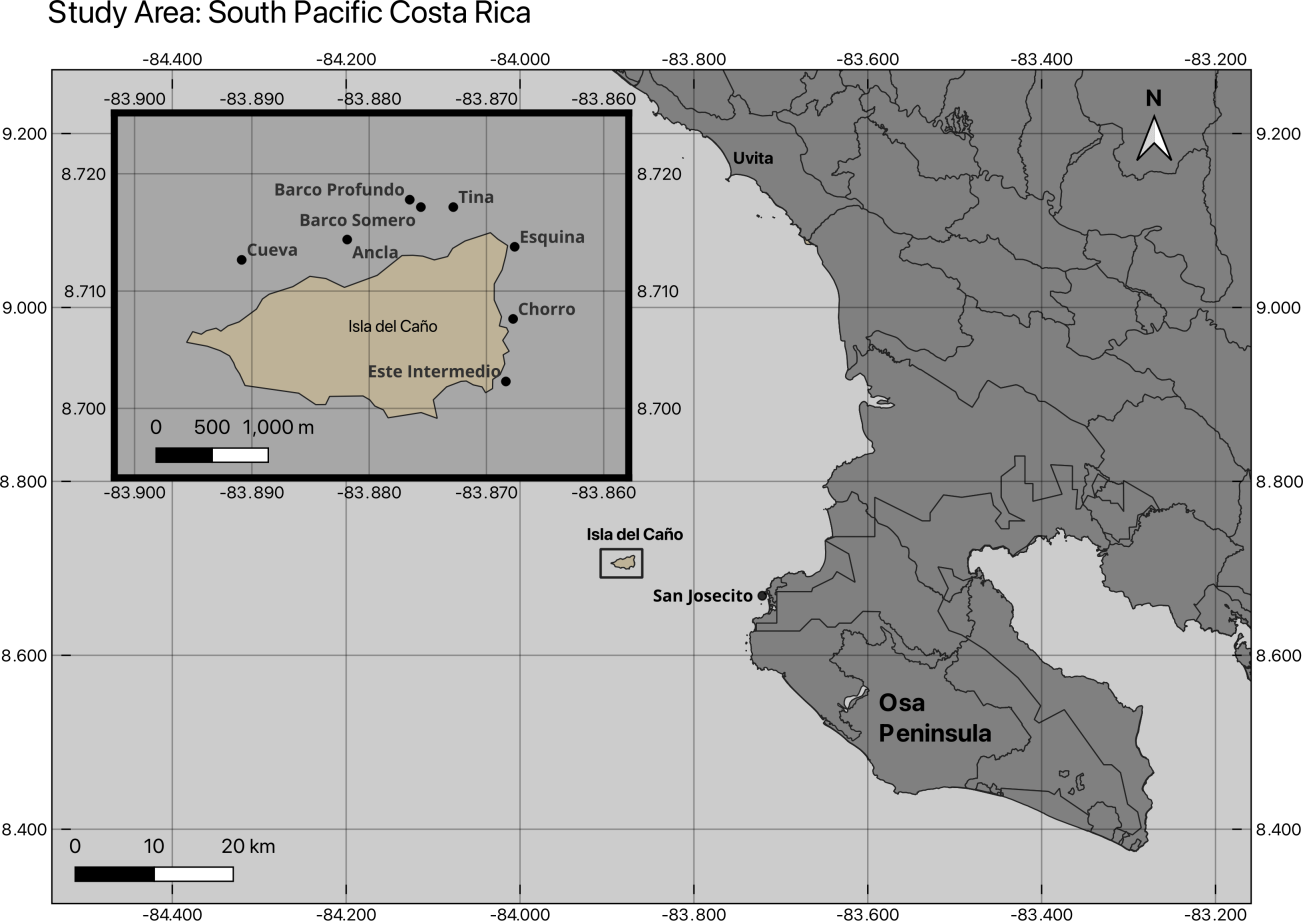
Map of coral community monitoring sites at Isla del Caño and northern Osa Peninsula. The location of the nine coral community sites surveyed.

Between 2019 and 2025, nine coral community sites were surveyed within the Reserva Biológica Isla del Caño (8°42′45.70″N 83°53′23.20″W) and on the northern coast of the Osa Peninsula within the Osa Conservation Area (ACOSA), Costa Rica (Table S1; Fig. 1). Of the investigated coral communities, which ranged in depth from <2 m to 16 m (Table S1), eight sites were surveyed for benthic cover and coral health between February 2024 and February 2025, including Ancla, Barco Somero, Barco Profundo, Cueva de Tiburones “Cueva”, Esquina, Este Intermedio, San Josecito, and Tina (Fig. 1). A ninth site, Chorro, was additionally surveyed for benthic cover in 2019, 2020 and both Chorro and Cueva in 2021 (Table S2). Landslides were observed at Isla del Caño in November 2025, with large sediment plumes still present in January 2025 (Fig. S2).

Benthic cover was recorded using different, but comparable, methods, including the categories: live coral (coral), sand, rubble, rock, CCA, recently dead coral (dead coral), turf algae, *Caulerpa*, cyanobacteria, and macroalgae. Recently dead coral was defined as an absence of tissue with recognizable skeletal features and absent or minimal overgrowth. *Caulerpa* was included as a distinct category because of its documented proliferation and impact on Costa Rican reefs (Fernández, 2007). For surveys in 2019, 2020 and 2021, three haphazardly-laid 10 m point intercept transects were used, recording the benthic cover category every 20 cm Sistema (Nacional de Áreas de Conservación (SINAC), 2021). Surveys conducted in February and March 2024 used 1 × 1 m quadrats to record the proportion of each benthic category over the full length of three 30 m (San Josecito) and three 10 m transects (Barco Somero, and Cueva; Table S2). For the remainder of the surveys, point and line intercept transects were conducted, recording substrate categories every 10 cm (in April 2024), and then the continuous 10 m line for three or six transects at each site (Tn = 270 transects; Table S2). Coral diversity, abundance and health were recorded from April 2024 using three or six 1 ×10 m belt transects, separated by 5 m. Corals were identified to genus, or species when possible, and categorized as healthy, pale, partially bleached (patches), mostly bleached (>50% of the colony) and bleached (whole colony). An individual coral colony was defined as having distinct area of tissue, meaning that multiple colonies could arise from a coral undergoing partial mortality. Benthic cover data were converted into proportions, enabling comparison of data collected using different survey methods. Total coral abundance, the abundance of each coral taxa, total bleaching prevalence and bleaching prevalence per species were calculated for each belt transect. Temperature loggers (ONSET HOBO MX2202) were programmed to record every 30 min and deployed at Ancla 15 m, Barco Profundo 16 m, Barco Somero 11 m and Tina (8 m), though remained temporarily and variably functional between April and August 2024.

Fieldwork was conducted under permit numbers: SINAC-ACOSA-DT-PI-R-006-2019, SINAC-ACOSA-DASP-PI-R-067-2021, SINAC-ACOSA-D-PI-R-013-2023, SINAC-ACOSA-DR-PI-R-024-2024.

### Data analysis

All analyses were conducted in R (R Core Team, 2021) and are summarized in the supplementary section (Table S4). Bayesian models were implemented in *brms* (Bürkner, 2017) using Stan (Carpenter et al., 2017) Hamiltonian Monte Carlo (HMC) sampling *via* the No-U-Turn Sampler (NUTS). Unless otherwise stated, models were fit with four chains of 4,000 iterations each (2,000 warm-up), using an adapt_delta of 0.99 for convergence and four cores for parallel computation. Model convergence was confirmed using R-hat values (≈1.00) and effective sample sizes (Bulk_ESS, Tail_ESS). Posterior predictive checks and leave-one-out cross-validation (LOO) were used to assess model fit and predictive accuracy. Proportion data (*e.g.*, coral cover, bleaching prevalence) were bounded between 0.0001 and 0.9999 to avoid boundary issues. Continuous predictors such as date were standardized using z-scores (*e.g.*, z_date) to improve numerical stability and allow comparability of effect sizes. Models involving proportional data were fitted using beta or zero-inflated beta (ZIB) distributions using a logit link for the mean and an identity link for zero inflation, where applicable. All *p*-values for non-Bayesian tests were Bonferroni-adjusted where appropriate.

### Coral bleaching prevalence and taxa-specific models

Site-level variation in bleaching prevalence was modelled using a Bayesian beta regression with logit link and a random intercept for site. Logit-scale intercept was used to estimate a baseline bleaching prevalence through inverse logit transformation. A multivariate ZIB regression model was used to analyze bleaching prevalence among different coral genera, incorporating structural zeros to account for sites where certain coral species were absent. The response variables included bleaching prevalence for coral taxon categories of *Pocillopora, Porites, Pavona, Psammocora*, and “Other” corals, with each modelled as a function of their respective structural zeros and those of other genera.

### Coral diversity

The Shannon Diversity Index was computed using total abundance of 13 coral taxa, including the Other category with the *vegan* package. Rows with missing species count values were removed. Due to significant deviations from normality and variance homogeneity (Shapiro–Wilk and Levene’s *p* < 0.05), Kruskal–Wallis tests were conducted to determine differences in taxa abundance among sites. Dunn’s *post-hoc* tests (Bonferroni-adjusted) were performed to identify pairwise differences between sites.

### Benthic composition shifts

Shifts in benthic composition were assessed using similarity percentage (SIMPER) analysis (*vegan*) conducted on “During Bleaching” (March to end of September 2024) and “After Disturbance” (December 2024 to February 2025) time periods. Ecologically relevant benthic variables were included; coral cover, macroalgae, turf, CCA, cyanobacteria, bleached coral and dead coral. SIMPER was performed at two levels: (1) across all sites combined to evaluate broad-scale compositional changes and (2) separately for each site to highlight site-specific patterns. Both analyses used 100 permutations to ensure robust estimates. Variables were ranked based on their contribution to compositional differences, and their mean before and after values were extracted to evaluate temporal trends. *P*-values were derived from permutation tests comparing within-group and between-group Bray–Curtis dissimilarities. These metrics identify the variables contributing most to observed shifts in community composition. To avoid artificial inflation of change, missing values were replaced with the mean for the time period at that site, or the global mean if the variable was entirely absent. Percentage change and *p*-values were used to identify the strongest drivers of composition shifts.

### Temporal trends in coral cover and benthic covariates

To assess temporal changes in coral cover, we used Bayesian ZIB regression models implemented in *brms*, with a logit link for the beta mean and identity link for the zero-inflation component. All models included site as a random intercept. Candidate models differed in how time was modelled (*e.g.*, linear *vs.* polynomial), and we compared their predictive performance using LOO, based on expected log predictive density (ELPD). The linear time model provided the highest predictive accuracy and was thus selected for inference. Based on this, we evaluated site-specific coral cover trends using simple linear regressions over scaled date (z_date), offering interpretable measures of change at each site. While more complex models could capture non-linear or heterogeneous dynamics, the linear model proved most appropriate given the temporal resolution and consistency across sites.

Temporal trends in benthic cover for algal groups (macroalgae, *Caulerpa*, turf algae, cyanobacteria, and CCA) were analyzed using Generalized additive models (GAMs) with beta error distribution and logit link. GAMs allowed flexibility in capturing potential nonlinear trends while controlling for site-level random effects. Penalized smoothing in mgcv allowed interpretability while avoiding overfitting. Models were fitted using REML, and diagnostics (residual checks, smooth term significance) were conducted to ensure model validity. A linear model was used to assess the relationship between coral cover and turf algae while accounting for site and temporal effects. Coral cover was modeled as a function of turf algae cover, site, and standardized time (z_date). The model was fitted using ordinary least squares regression, with model assumptions checked *via* residual diagnostics.

### Coral cover and local temperature

HOBO logger data from four locations: Barco Profundo, Barco Somero, Tina, and Ancla was used to evaluate differences in local temperature conditions across sites. Raw temperature data were processed to calculate monthly mean, maximum, and minimum temperatures per site. All values were aggregated at monthly resolution after cleaning and aligning timestamps to ensure maximum overlap in sampling dates among sites. A one-way ANOVA was conducted separately for mean, max, and min monthly temperature data to test for differences among sites. Tukey’s Honest Significant Difference (HSD) *post hoc* tests were performed to identify pairwise differences. All comparisons were adjusted for family-wise error rate, and significance was determined at *α* = 0.05. A beta regression model was used to analyze the effects of temperature and site on coral cover. The initial model included mean, minimum, and maximum temperature as predictors but was refined based on statistical significance and model performance. Minimum and maximum temperature were removed due to their non-significant effects (*p* = 0.585 and *p* = 0.429, respectively), resulting in a final model with mean temperature and site as predictors. The beta regression framework, with a logit link function for the mean and an identity link for the precision parameter (*φ*), was chosen to account for heteroscedasticity and the bounded distribution of coral cover. Maximum likelihood estimation (BFGS optimization) was applied, and model performance was assessed using pseudo R^2^.

### Temporal trends in coral taxa

Temporal trends in the proportional cover of *Pocillopora, Porites, Pavona, Psammocora*, and Other coral were modelled using a multivariate beta regression model with random slopes. This allowed proportional data to be modelled while accounting for variation in coral cover (random intercept) and divergent temporal trends among sites (random slope for z_date). Alternative models were tested to determine the best structure. Model comparison showed that the random slopes model provided a better balance between complexity and interpretability than more parameter-rich interaction models, which increased uncertainty without improving fit. Models were implemented in *brms*, and convergence was confirmed through standard diagnostics.

### Multivariate benthic analysis

Principal component analyses (PCAs) were performed to explore multivariate gradients of key benthic parameters. An initial, full model PCA was conducted including biological and benthic variables, as well as total coral cover and the abundance each of *Pocillopora* spp., *Porites* spp., *Pavona* spp., *Psammocora* spp., and Other coral, and environmental parameters (Supplementary File). Variables with minimal influence on components were then removed from the analysis, including pH, salinity, phosphate, and oxygen. In the first two PCA analyses, *Caulerpa* and dead coral contributed to PC1; however, they were not observed in the most recent surveys and thus were absent from the final PCA. This absence reflects an ecological shift rather than a methodological change, indicating that these components were no longer present at detectable levels during the final sampling period.

PCAs were run on three datasets: (i) During Bleaching (all survey data up to July), (ii) After Bleaching (August to December 2024), and (iii) the Latest dataset (January or February 2025) for all sites, to capture potential shifts post-La Niña rains and landslide events. Each PCA used standardized variables (mean = 0, SD = 1), and *prcomp* in R was employed with centring and scaling. Zero-variance variables were removed, and incomplete cases were filtered out. PCA loadings were used to identify which benthic or environmental variables contributed most strongly to disparities among sites. To assess temporal trends in benthic community composition, PCA loadings were extracted and standardized to facilitate direct comparison and site scores were compared across the three time periods. Key benthic variables structuring PC1 were examined to identify benthic community composition shifts. All variables were transformed to z-scores (mean = 0, SD = 1) before PCA to prevent biases from differences in magnitude or measurement units. Only variables present in all three PCA results were included, excluding those filtered out due to low variance or collinearity. PC1 orientations were aligned across time periods to ensure consistent interpretation, with eigenvectors adjusted by multiplying by -1 when necessary.

### Ecological recovery feasibility index

Composite, multi-metric indices are well established in restoration science, *e.g.*, expert-driven priority scores for forests (Orsi, Geneletti & Newton, 2011) and mangroves (Su et al., 2022), and leading coral-reef restoration guides recommend site ranking, suggesting a call for analogous tools (Goergen et al., 2025; Shaver et al., 2020). However, no existing index integrates site-specific benthic data with data-driven weights and continuous scoring of both positive (coral cover, CCA, diversity; Nyström et al., 2008; Tebben et al., 2015) and negative (turf algae, cyanobacteria, bleaching; Ford et al., 2018) drivers. We designed the Ecological Recovery Feasibility Index (ERFI) as an initial, generalized screening tool to rank reefs by their baseline benthic-community conditions and identify those most likely to support natural recovery. The ERFI therefore produces a transparent, replicable restoration feasibility score.

To obtain PCA-derived weights we performed a PCA on six benthic variables (live coral cover, CCA cover, turf-algal cover, cyanobacterial cover, bleaching prevalence, Shannon diversity) using the Latest Data (January–February 2025). For each variable j, we extracted its loadings *lj*_1_, *lj*_2_, *lj*_3_ on principal components 1–3 and summed their absolute values to obtain:

\begin{eqnarray*}{L}_{j}= \left\vert {l}_{ \left\{ j1 \right\} } \right\vert + \left\vert {l}_{ \left\{ j2 \right\} } \right\vert + \left\vert {l}_{ \left\{ j3 \right\} } \right\vert . \end{eqnarray*}

Expert direction weights, *Dj,* were assigned to each variable based on ecological effect:

\begin{eqnarray*}{D}_{j}= \left\{ \begin{array}{@{}c@{}} \displaystyle +4~if~j=coral;\\ \displaystyle +2~if~j=CCA~or~diversity;\\ \displaystyle -2~if~j=cyanobacteria;\\ \displaystyle -2~if~j=bleaching;\\ \displaystyle -3~if~j=turf~algae \end{array} \right\} . \end{eqnarray*}

For each site* i* and variable *j*, we computed the z-score:

\begin{eqnarray*}{z}_{ \left\{ ij \right\} }= \frac{ \left( {x}_{ \left\{ ij \right\} }-{\overline{x}}_{j} \right) }{{s}_{j}} \end{eqnarray*}

where xij is the observed value, and x^−^j and sj are the mean and standard deviation of variable j across all sites.

To calculate composite weights and raw ERFI, we multiplied the PCA weight and the direction weight to obtain:

\begin{eqnarray*}{C}_{j}={L}_{j}\times {D}_{j} \end{eqnarray*}

and then summed across variables to compute each site’s raw score:

\begin{eqnarray*}ERF{I}_{i}=\sum _{j} \left( {C}_{j}\times {z}_{ \left\{ ij \right\} } \right) . \end{eqnarray*}

Finally, we completed a normalization step [0, 1] to facilitate interpretation. We rescaled the raw scores by:

\begin{eqnarray*}ERF{I}_{i}\ast = \frac{ \left( ERF{I}_{i}-\min \nolimits \left( ERF{I}_{i} \right) \right) }{ \left( \max \nolimits \left( ERF{I}_{i} \right) -\min \nolimits \left( ERF{I}_{i} \right) \right) } . \end{eqnarray*}

A worked numeric example of the above steps is provided in Table S3.

## Results

### SST at Isla del Caño

**Figure 2:**
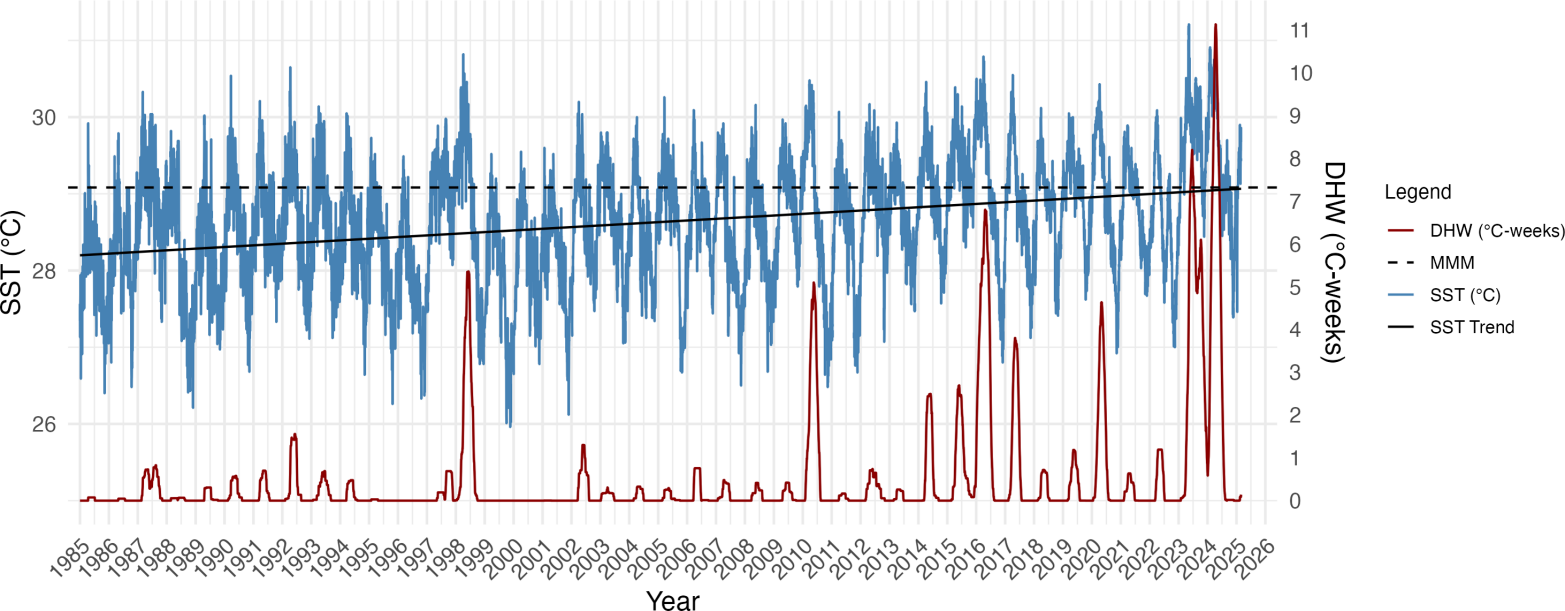
Daily sea surface temperature (SST), maximum monthly mean (MMM), and Degree Heating Weeks (DHW) for Isla del Caño at 5 km resolution from 1985 to 2025. DHW was calculated using a bleaching threshold of MMM + 0.5 °C, based on a 12-week rolling sum, following Lachs et al. (2023), to enhance sensitivity to localized thermal stress. The MMM (29.08 °C) was derived from the climatological period 1985–1990 and 1993, excluding volcanic anomaly years. The black line represents the linear regression fit to the SST data, with an estimated warming rate of 0.23 °C per decade (SST = a + 0.0023 × year). Peaks in DHW are especially notable during El Niño years (1998, 2016, and 2023–24), with the 2023–24 event producing the most intense thermal stress observed in the record.

SST increased significantly over the 40-year period (1985–2025) at Isla del Caño (*R*^2^ = 0.095; *F*_(1,14642)_ = 1544, *p* = 2.2e−16; Fig. 2) with an MMM of 29.08 °C based on the highest monthly mean SST (1985–1990 and 1993). The estimated rate of SST increase was approximately 0.23 °C per decade, equating to a 0.92 °C warming over the 40-year period (Fig. 2). Incorporating the ONI into the regression model significantly improved the fit (*R*^2^ = 0.28; *F*_(2,14606)_ = 2872, *p* = 2.2e−16), confirming that ENSO as a strong predictor of SST and modulator of SST anomalies. El Niño periods are visible in the Isla del Caño SST record, with peaks or clustered peaks in SST and DHW most prominent in 1997–98, 2010, 2014–17, 2020 and 2023–24 (Fig. 2). The triple peaks of DHW during the 2023–24 El Niño reached 1.6-fold higher than the peak of the 2016 event, and 2-fold greater than the peak of 1998 (Fig. 2). The highest SST was also recorded during the 2023–24 El Niño, reaching 31.2 °C, with peaks in 1998 and 2016 remaining below 31 °C (Fig. 2).

Linear regression models indicate that the annual frequency of days breaching the bleaching threshold (MMM + 0.5 °C) has significantly increased through time (Fig. S1), with an estimated additional 2.07 bleaching days per year (*F*_(1,39)_ = 12.94 ; *p* = 0.0009; *R*^2^ = 0.23). Conversely, that the frequency of cool days has significantly decreased, by approximately 0.72 days per year (*F*_(1,39)_ = 17.62 ; *p* = 0.0002; *R*^2^ = 0.29). Years prior to 2012 predominantly experienced frequent cool days, including in El Niño years when warm (bleaching) days were also frequent, including 1998 and 2010 (Fig. S1). After 2012, the frequency of cool days is markedly less, with no substantial cool periods occurring since 2021. Warm days were most frequent in 2023, amounting to two thirds of the year with no cool days (Fig. S1).

### Local impacts, bleaching prevalence and coral susceptibility

**Figure 3:**
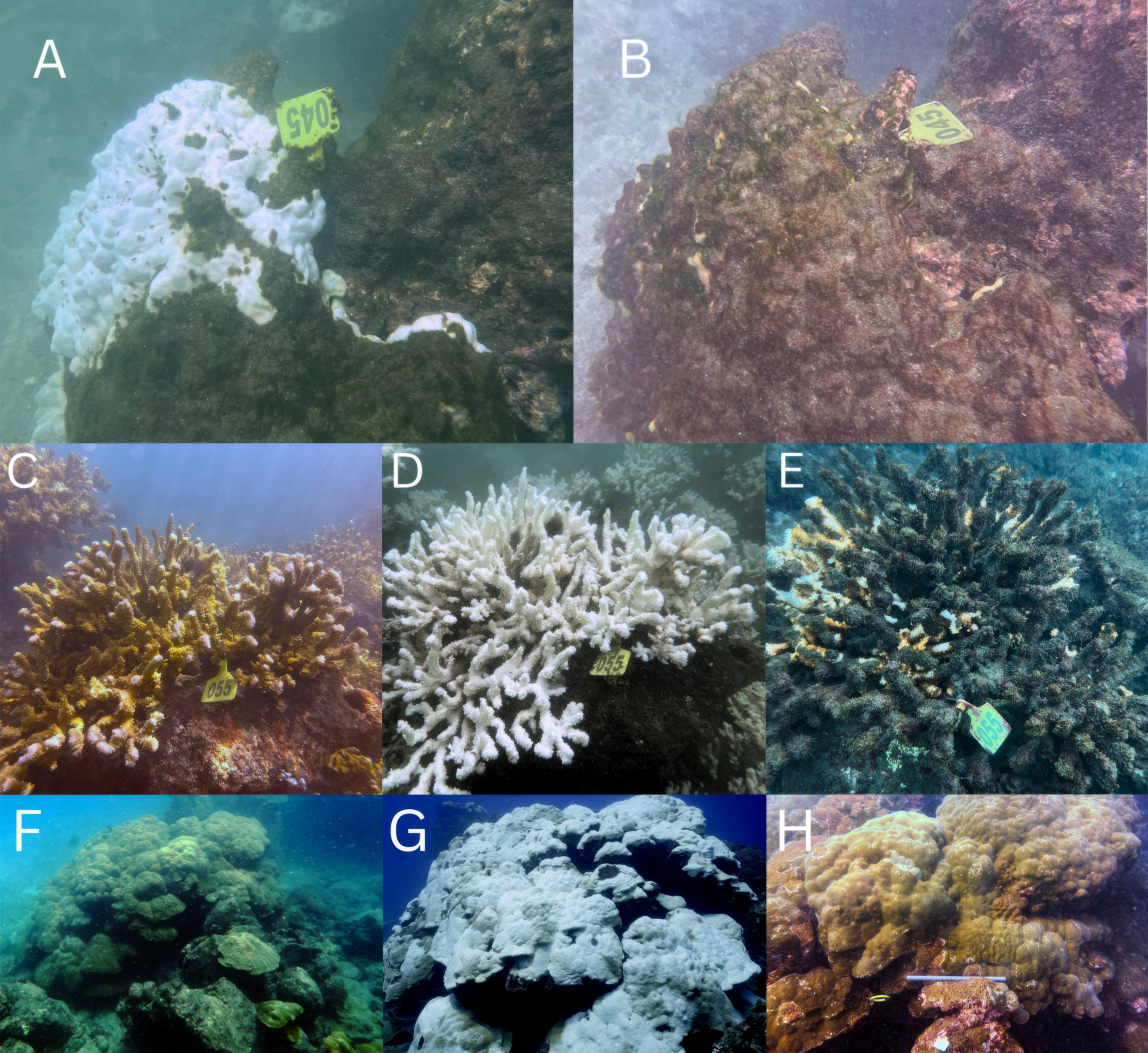
Visual evidence of bleaching, mortality, and recovery in *Porites lobata* and *Pocillopora* spp. at Isla del Caño. (A–B) *Porites lobata* colony at San Josecito (∼1 m depth), photographed on 22 February 2024 (A) and 30 May 2024 (B), showing bleaching and subsequent mortality. (C–E) *Pocillopora* spp. colony at San Josecito (∼1 m depth), observed on 28 January 2024 (C), 22 February 2024 (D), and 30 May 2024 (E), showing progression from healthy to bleached to partially dead. (F–H) Time series of a *Porites lobata* colony at Barco Somero, photographed as healthy in 2019 (F), severely bleached in 2023 (G), and recovered but with partial tissue loss in 2025 (H).

Severe, mortality-inducing coral bleaching was observed at Isla del Caño from 2023 and throughout 2024 (Fig. 3), prior to the study period (Table S1). Numerous corals bleached and were observed to progress to a state of partial or complete mortality (Figs. 3A–3E), suggesting that the 2024 to 2025 survey data span the tail-end of an extreme bleaching event (Fig. 2). Some corals were photographed severely bleached and subsequently recovered (Figs. 3F–3H). Additionally, in late November 2024 during the rainy season, a series of landslides occurred on Isla del Caño (Figs. S2A and S2B), with a significant amount of sediment washed onto reef sites (particularly Cueva and Ancla; Fig. S2C). During this time the Biological Reserve was closed to the public due to unsafe conditions, and it is unclear exactly when the landslides occurred and the immediate extent of the sediment plumes. Crown-of-thorns starfish were also observed sporadically at several sites, including predating on massive coral species (Fig. S3).

**Figure 4:**
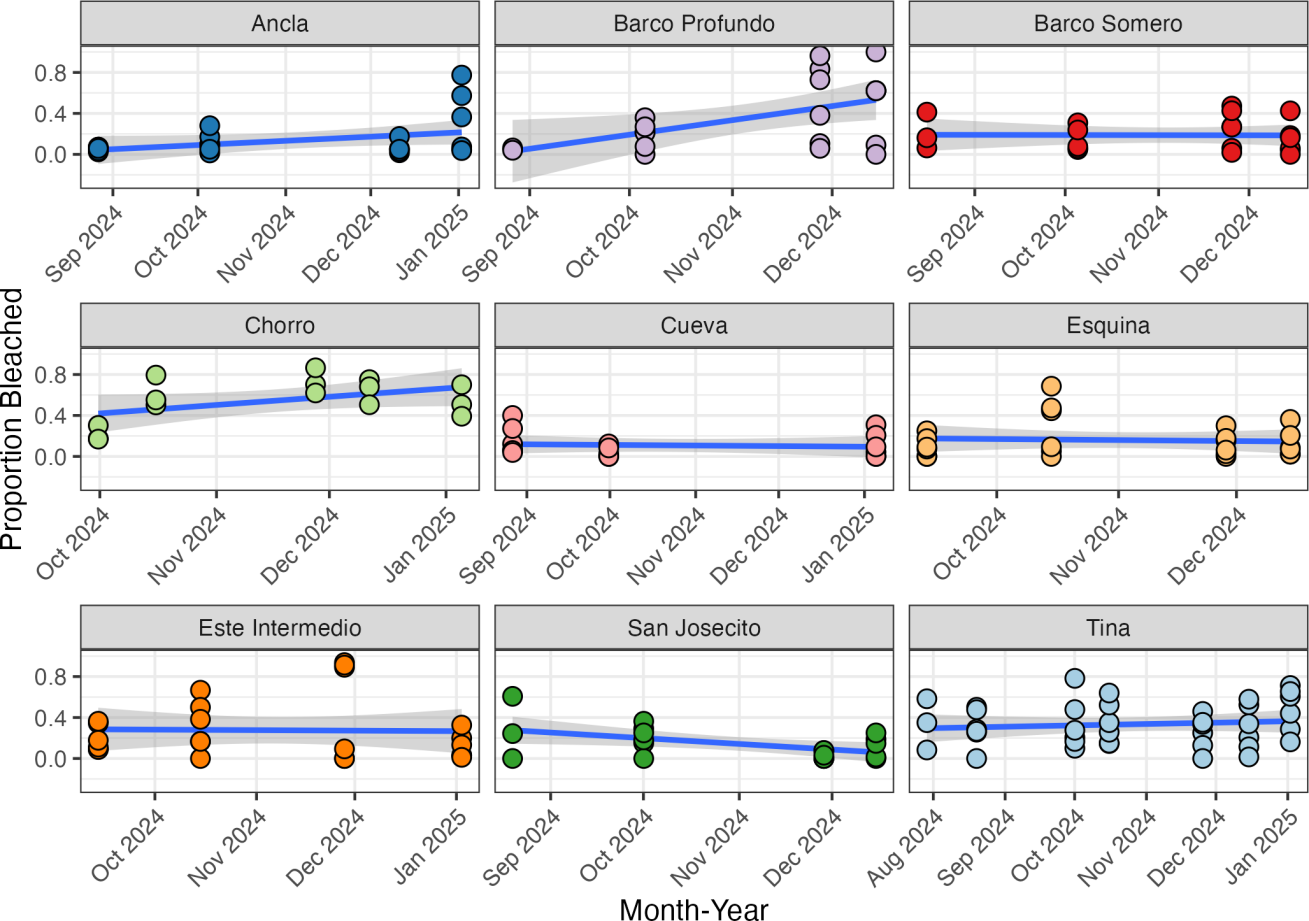
The proportion of bleached corals at each site recorded during the study period. Each data point represents the mean proportion of bleached colonies recorded per individual transect at a specific site and time point. The blue line indicates the fitted regression line from a linear model, with the shaded area representing the 95% confidence interval of the fitted values.

There was a baseline bleaching prevalence of approximately 23% across all surveys, as calculated through logit transformation (−1.17; 95% CI [−1.54 to −0.79]), with significant variation in bleaching prevalence among sites (beta regression with logit link; SD 0.48; 95% CI [0.22–0.91]) and moderately low within site variability (phi 1.94; 95% CI [1.61–2.32]). Ancla, Barco Profundo, and Chorro demonstrated increasing bleaching prevalence through time (Fig. 4), whilst decreasing prevalence was observed at San Josecito. The remaining sites, Barco Somero, Tina, Cueva, Este Intermedio and Esquina had approximately constant bleaching prevalence through time (Fig. 4).

Baseline bleaching prevalence varied among coral genera. Model (ZIB) estimates indicate that *Pocillopora* spp. exhibited the highest bleaching prevalence at 33.9%, followed by *Porites lobata* (24.0%), *Psammocora* spp. (15.6%), and *Pavona* spp. (10.1%), while Other corals exhibited minimal bleaching at just 0.25% (Table 1). The estimated standard deviation of site-level variation in bleaching prevalence was highest for *Porites lobata* (SD = 0.19), suggesting greater among-site variability than other taxa, and was lowest for Other coral (SD = 0.08). *Pocillopora* spp.*, Psammocora* spp. and *Pavona* spp. exhibited intermediate variability. The dispersion parameter (phi) estimates suggest moderate consistency in bleaching prevalence for most genera, while *Pavona* spp. showed moderate dispersion and Other coral had high dispersion, indicating close to zero bleaching in this group across all sites (Table 1). Zero-inflation was negligible across all coral taxa (Table 1), indicating that observed zeros were consistent with model expectations and that zero-inflated components were not necessary.

### Coral diversity

A total of 12 coral taxa were observed and identified at Isla del Caño, including *Pocillopora spp., Porites lobata, Pavona clavus, Pavona gigantea, Pavona varians, Pavona maldiviensis, Pavona chiriquiensis, Pavona frondifera, Psammacora stellata, Psammacora profundacella, Tubastraea coccinea* and *Gardineroseris planulata*. *Pocillopora* spp. and *Porites lobata* were present at all sites, and *Pavona clavus* and *Pavona gigantea* were present at all sites except Este Intermedio and San Josecito, respectively. *Gardineroseris planulata* was the rarest coral taxon, observed only at Ancla, Barco Somero and Cueva (Table S5).

**Figure 5:**
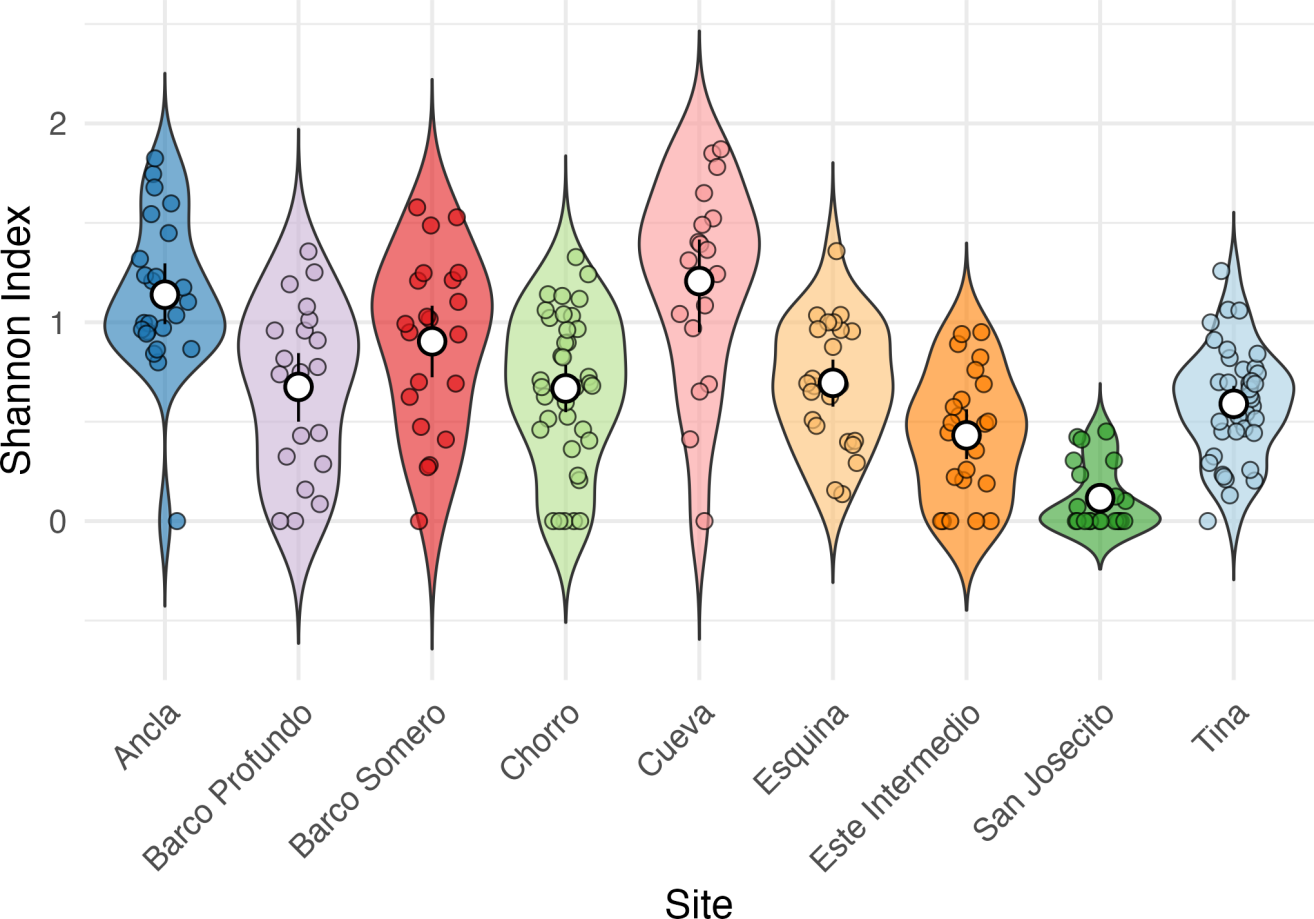
Shannon Index of coral species diversity among reef sites for the full study period. Ancla *n* = 18, Barco Profundo *n* = 24, Barco Somero *n* = 24, Chorro *n* = 48, Cueva *n* = 18, Esquina *n* = 24, Este Intermedio *n* = 18, San Josecito *n* = 27, Tina *n* = 33.

Shannon Diversity Index varied significantly among sites (Kruskal-Wallis test: *χ*^2^ = 92.623, *df* = 8, *p* < 2.2*e* − 16; Fig. 5), with Cueva exhibiting the highest mean Shannon Index (1.28 ± 0.43), followed by Ancla (1.14 ± 0.39) and Barco Somero (0.90 ± 0.44). San Josecito had the lowest diversity (0.12 ± 0.16), with Este Intermedio also showing relatively low values (0.43 ± 0.31). *Post-hoc* Dunn’s test confirmed significant differences among sites (Table S6). For instance, Cueva had significantly higher diversity than San Josecito (p adj. = 6.30E−12), Este Intermedio (p adj. = 0.000002), and Esquina (p adj. = 0.02). Ancla also had significantly higher diversity than San Josecito (p adj. = 2.94E−12) and Este Intermedio (p adj. = 2.99E−06) (Table S6).

**Table 1:** Bleaching prevalence and variability estimates for coral genera from the zero-inflated beta (ZIB) model (95% CI).

Coral genus	Bleaching prevalence (%)	Site-level variation (SD)	Dispersion (phi)	Zero inflation (zi)
*Pocillopora* spp.	33.9 (24.4–44.9)	0.13 (NA)	0.82 (0.68–0.97)	0.00 (0.00–0.02)
*Porites lobata*	24.0 (16.3–34.0)	0.19 (0.01–0.51)	1.08 (0.90–1.27)	0.00 (0.00–0.02)
*Psammocora* spp.	15.6 (10.2–22.6)	0.10 (NA)	0.92 (0.73–1.14)	0.00 (0.00–0.02)
*Pavona* spp.	10.1 (6.7–15.2)	0.10 (NA)	1.61 (1.21–2.08)	0.00 (0.00–0.02)
Other corals	0.25 (0.16–0.37)	0.08 (0.00–0.24)	105.63 (75.51–140.02)	0.00 (0.00–0.02)

### Community composition change

The SIMPER analysis identified key contributors to benthic composition changes across sites between the “During Bleaching” (March to August 2024) and “After Disturbance” (December 2024 to February 2025) periods (Tables 2 and 3). Turf algae and dead coral contributed most to overall community dissimilarity, with turf increasing by 70.62% (*p* = 0.010) and dead coral decreasing by 81.30% (*p* = 0.010; Table 2). Coral cover declined significantly by 40.42% (*p* = 0.010), from a mean of 19.33% to 11.46%. CCA increased modestly by 13.0%, though not significantly (*p* = 0.347). Macroalgae and cyanobacteria declined by 34.96% (*p* = 0.069) and 74.37% (*p* = 0.010), respectively. Bleaching also contributed to dissimilarity, increasing by 35.30%, but was not significant (*p* = 0.881).

**Table 2:** Key contributors to overall benthic composition change (SIMPER).

Variable	Average contribution	Consistency ratio	Mean before	Mean after	Cumulative contribution	% Change	*P*-value
Turf	0.1598	1.466	0.337	0.575	0.332	70.62	0.0099
Bleached	0.1101	1.282	0.192	0.26	0.561	35.42	0.8811
Coral	0.0821	1.094	0.193	0.115	0.732	−40.41	0.0099
CCA	0.062	0.785	0.094	0.106	0.861	12.77	0.3465
Dead coral	0.0455	0.571	0.093	0.017	0.955	−81.72	0.0099
Macroalgae	0.0125	0.532	0.017	0.011	0.981	−35.29	0.0693
Cyanobacteria	0.009	0.563	0.016	0.004	1.0	−75.0	0.0099

**Table 3:** Key contributors to substrate change at each site, based on significance (SIMPER).

Site	Variable	Cumulative contribution	% Change	*P*-Value
Ancla	Turf	0.42	70.78	0.069
	CCA	0.607	195.96	0.455
	Coral	0.783	−53.99	0.04^*^
Barco Profundo	Turf	0.445	203.81	0.01^*^
	Bleached	0.735	676.25	0.04^*^
	CCA	0.821	211.5	0.257
Barco Somero	Macroalgae	0.279	−99.59	0.01^*^
	Turf	0.476	29.25	0.802
	Bleached	0.638	−13.76	1.0
Chorro	Turf	0.31	38.01	0.267
	Bleached	0.56	42.05	0.871
	Coral	0.704	28.07	0.436
Cueva	Turf	0.425	91.87	0.01^*^
	CCA	0.623	−22.71	0.693
	Bleached	0.762	−49.54	0.02^*^
Esquina	Turf	0.367	44.12	0.02^*^
	Coral	0.585	−50.55	0.01^*^
	Bleached	0.79	83.73	0.693
Este Intermedio	Turf	0.313	59.57	0.257
	Bleached	0.553	54.61	0.97
	Dead coral	0.711	−97.64	0.01^*^
San Josecito	Turf	0.399	344.03	0.03^*^
	Dead coral	0.748	−99.75	0.01^*^
	Bleached	0.915	−57.96	0.04^*^
Tina	Turf	0.329	62.26	0.277
	Coral	0.621	−48.02	0.01^*^
	Bleached	0.887	9.66	0.663

**Notes.**

* indicates statistical significance.

Site-level patterns revealed strong declines in coral cover at several locations (Table 3). Tina experienced a 52.44% decline (*p* = 0.010), while Ancla and Esquina showed declines of 46.0% (*p* = 0.040) and 50.56% (*p* = 0.010), respectively. Coral cover also declined at Barco Somero (−8.4%) and Este Intermedio (−45.34%), though these were not significant (Table 3). Turf algae increased at Ancla, Barco Profundo, Chorro, Cueva, and Esquina, with changes ranging from 43.2% to 336.7%, but was only significant at Cueva (*p* = 0.010), Barco Profundo (*p* = 0.010), and San Josecito (*p* = 0.030; Table 3; Fig. S3). Macroalgae declined at most sites and was a notable contributor to benthic change at Barco Somero, Tina, Barco Profundo, San Josecito, and Este Intermedio (Table 3; Fig. S4). Recently dead coral at San Josecito declined by 99.75% (*p* = 0.010) and similarly at Cueva (−97.1%, *p* = 0.010), Chorro (−29.8%, *p* = 0.277), and Este Intermedio (−97.64%, *p* = 0.010). The mean percentage coral cover after disturbance was lowest at Barco Profundo (3.52%) and highest at Chorro (16.86%) (Table S7). Four sites had less than 10% coral cover after disturbance (Cueva, Barco Profundo, Ancla, and San Josecito), while Esquina had 10.59% and Este Intermedio 11.12%.

### Temporal changes in coral cover

**Figure 6:**
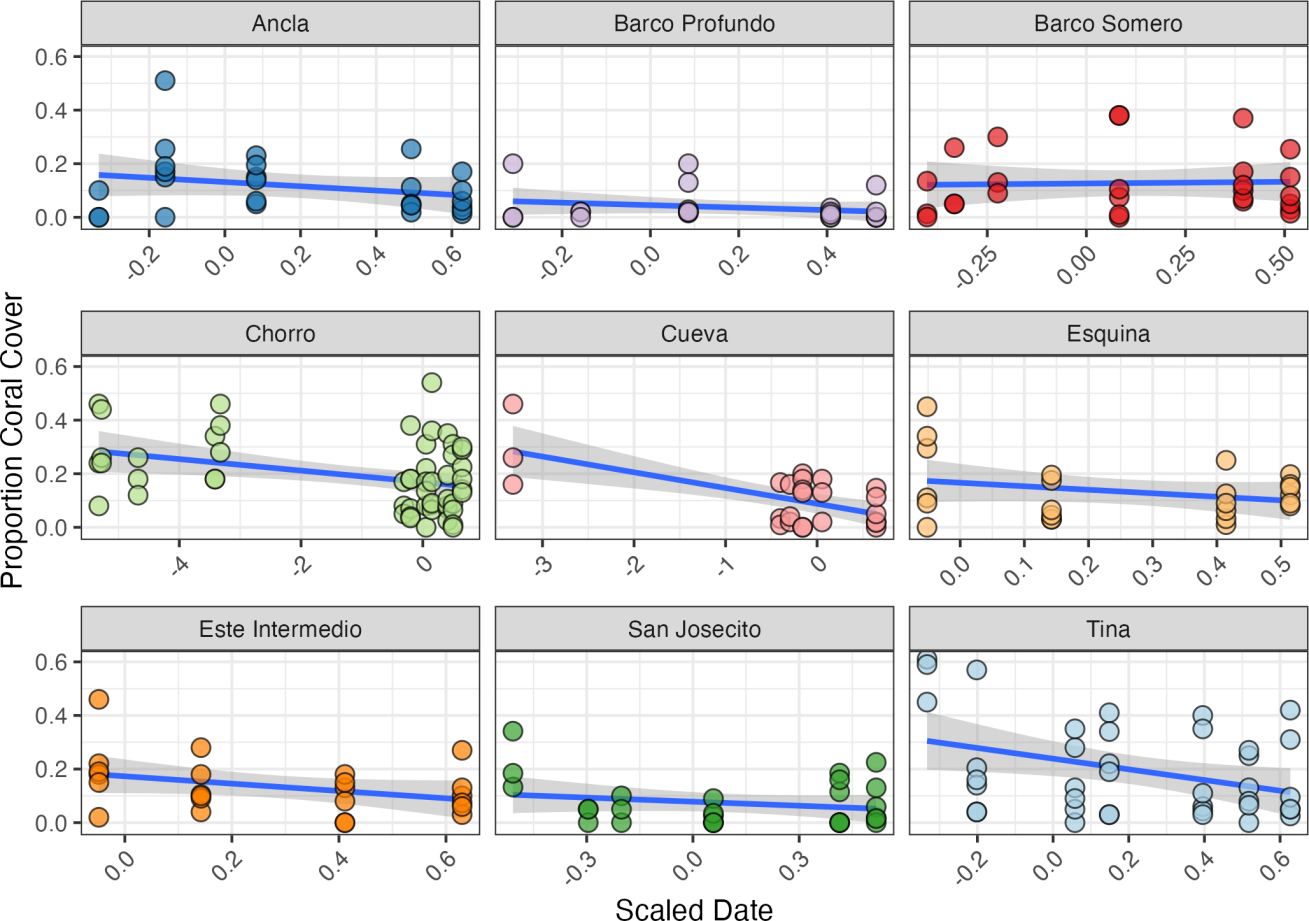
Proportion of coral cover at each site through time. The blue line represents the fitted regression line from the linear model, and the shaded area around the line indicates the 95% confidence interval of the fitted values. Ancla *n* = 24, Barco Profundo *n* = 24, Barco Somero *n* = 24, Chorro *n* = 54, Cueva *n* = 26, Esquina *n* = 24, Este Intermedio *n* = 24, San Josecito *n* = 27, Tina *n* = 39. Linear trend lines are provided for visual interpretation only; formal analyses of coral cover trends were conducted using a Bayesian zero-inflated beta model.

The initial proportion of coral cover varied among sites, with Tina exhibiting the highest mean cover at 55% (SD ± 0.09, *n* = 3) in mid-April 2024, and Barco Profundo the lowest, with less than 1% (SD ± 0.115, *n* = 3; Fig. 6). Bayesian ZIB regression model revealed a general decline in coral cover over time. The best-supported model included a fixed linear effect of time and a random intercept for site. This model estimated a significant negative linear effect (Estimate = −0.19, 95% CI [−0.29 to −0.08]), indicating a consistent decline in coral cover across the study period. The overall baseline coral cover was estimated at approximately 11.4% (Intercept = −2.05, 95% CI [−2.45 to −1.66]), with moderate variation among sites (SD = 0.53, 95% CI [0.28–0.98]). There was no evidence of substantial zero-inflation (zi = 0.00, 95% CI [0.00–0.01]), suggesting that complete coral absence was rare. Linear regressions exploring site-level variability in coral trajectories highlighted the strongest significant decline in coral cover at Cueva (slope = −0.059, SE = 0.014, *p* < 0.001; Fig. 6), followed by Chorro (−0.022, SE = 0.008, *p* = 0.009) and Tina (−0.199, SE = 0.085, *p* = 0.024). Coral cover declines at Este Intermedio (−0.137, *p* = 0.096), Esquina (−0.131, *p* = 0.204), Ancla (−0.079, *p* = 0.204), Barco Profundo (−0.046, *p* = 0.277), San Josecito (−0.050, *p* = 0.287) and Barco Somero (−0.012, *p* = 0.858) were not statistically significant.

### Temporal trends in algal cover

GAMs used to assess temporal trends in algal cover demonstrated a significant non-linear temporal trend in macroalgae (e*df* = 5.012, *p* = 0.0055) with site-level variability (e*df* = 5.299, *p* = 0.0013), with an adjusted R^2^ of 0.0139. *Caulerpa* exhibited a weaker temporal pattern (e*df* = 1.746, *p* = 0.0113), though site effects were negligible (*p* = 0.8621). Turf algae displayed a strong temporal trend (e*df* = 1.665, *p* < 0.0001) without a site effect (*p* = 0.88), explaining 33% of deviance. Cyanobacteria and CCA showed no significant temporal trends (*p* = 0.187 and *p* = 0.48, respectively), although CCA exhibited significant site-level variation (e*df* = 7.120, *p* < 0.0001).

A linear model exploring the relationship between coral cover and turf algae demonstrated a significant negative association (*β* = −0.131, SE = 0.039, t = −3.40, *p* = 0.00081), indicating that higher turf algae cover corresponded with lower coral cover. Site effects were significant at Barco Profundo (*β* = −0.087, SE = 0.034, t = −2.53, *p* = 0.012) and Tina (*β* = 0.059, SE = 0.030, *t* = 1.98, *p* = 0.049), highlighting site-specific differences in coral cover. Temporal trends were not significant (*β* = −0.035, SE = 0.033, t = −1.08, *p* = 0.282; Fig. S5).

### Site temperature and coral cover

Mean monthly temperatures differed significantly among the four sites (ANOVA: *F*_(3,10)_ = 8.39, *p* = 0.0044; Fig. S6), whereas no significant differences were detected for maximum (*F*_(3,10)_ = 0.281, *p* = 0.838) or minimum temperature (*F*_(3,10)_ = 2.95, *p* = 0.085). *Post hoc* comparisons revealed that Tina had significantly higher mean temperatures than both Ancla (difference = +1.12 °C, *p* = 0.023) and Barco Profundo (+1.63 °C, *p* = 0.0035; Table S8). No other pairwise comparisons were statistically significant. Coral cover was significantly influenced by mean temperature and varied across sites. Mean temperature had a positive effect (Estimate = 0.877, SE = 0.275, *z* = 3.187, *p* = 0.0014), indicating higher coral cover at warmer sites. The model explained 32.91% of the variation in coral cover (Pseudo *R*^2^ = 0.3291), suggesting that while temperature influenced coral cover, site-specific factors played a substantial role in shaping spatial patterns. The precision parameter (*φ* = 5.542, SE = 1.058, *p* < 0.0001) indicated low dispersion.

### Temporal trends in the proportion cover of each coral taxa

The multivariate beta regression model with random slopes indicated variability in coral cover trends among sites. Posterior estimates showed no strong overall trends in the proportion cover of each coral taxa through time, though site-level differences may be driving variation in coral composition over time, as indicated by site-level variation in coral proportion changes (Table S9; Fig. S7).

### Shift from coral to algal dominance

**Figure 7:**
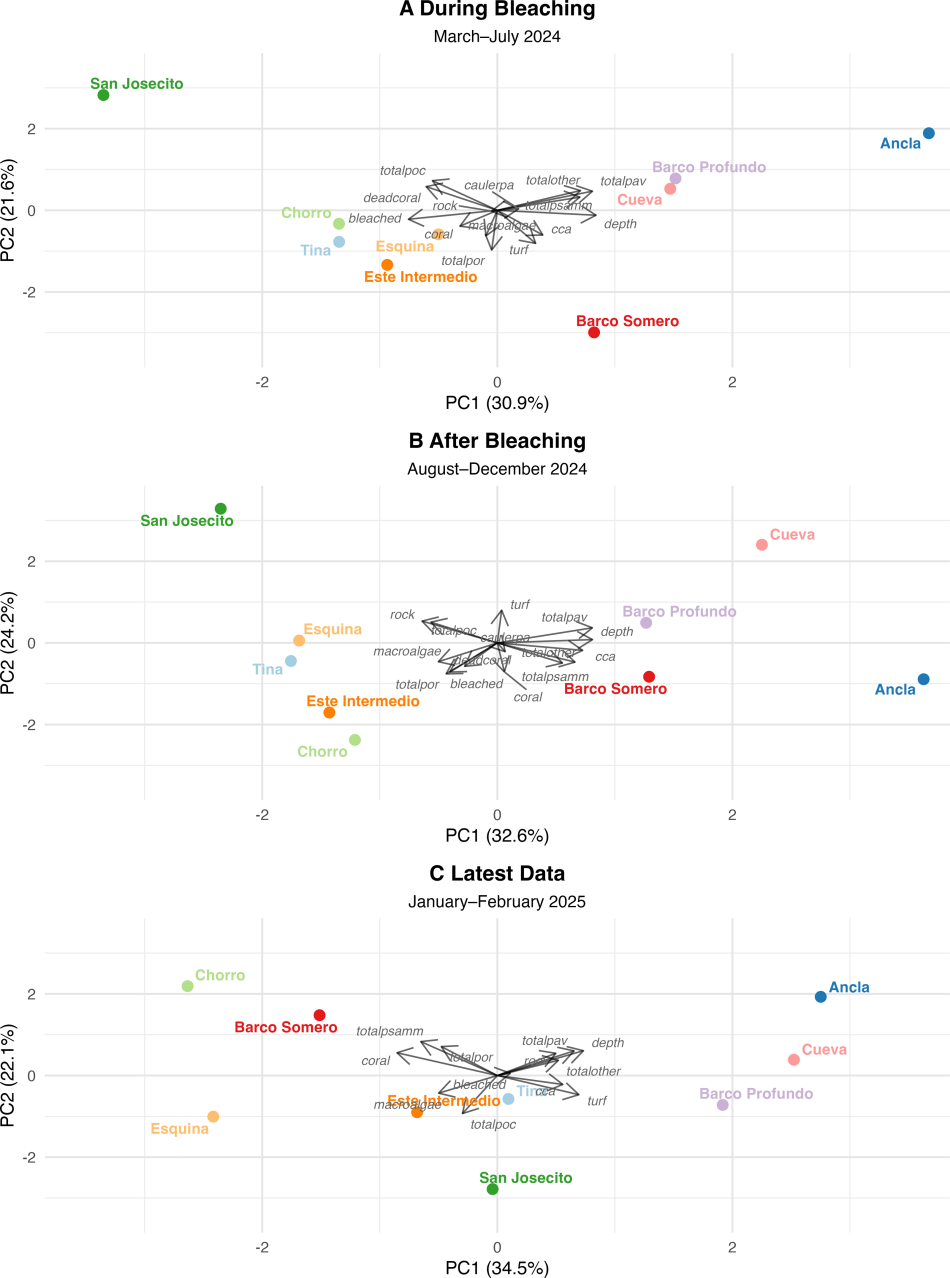
Principal component analysis (PCA) of reef site composition across different time periods. (A) During Bleaching: March to July 2024, (B) After bleaching: August to December 2024 and (C) Latest data: January to February 2024. Arrows indicate PCA loadings, representing the contribution of environmental and benthic variables to site differentiation.

PCA were used to assess changes in reef structure across three sequential time points (Tables S10 and S11): During bleaching (March to July 2024), After bleaching (August to December 2024), and the Latest period (January to February 2025). Each PCA was interpreted independently and captured a high proportion of total variance in reef composition and collectively indicate shifts in community structure from coral dominance toward turf algae dominance at most sites (Fig. 7). While some sites have stabilized with remaining coral cover, others have continued to degrade, with San Josecito experiencing a near-total collapse. *Pocillopora* spp. was more predominant at shallow sites and was associated with high bleaching prevalence (Fig. 7). *Pavona* spp. and *Psammocora* spp. were initially associated with deeper reefs (Cueva and Ancla; Fig. 7A), though lost prominence as turf algae and bleaching increased (Figs. 7A and 7C). Similarly, *Porites* spp., while less strongly associated with deep sites, also reduced in dominance as turf and bleaching increased (Fig. 7). The absence of dead coral by the Latest Data PCA (Fig. 7C) suggests that sites initially characterized by recent mortality transitioned to a different state, likely dominated by algal overgrowth.

### During bleaching (March to late July 2024)

The first four principal components (PCs) of survey data from March to late July 2024 explained 80.6% of the total variance, with PC1 (30.9%) and PC2 (21.6%) as the dominant axes. PC1 primarily reflected a depth-related gradient, with deeper sites associated with *Pavona* spp., *Psammacora* spp., and Other coral (positive loadings), contrasting with sites experiencing high bleaching and dead coral. Ancla, Cueva, and Barco Profundo aligned with deeper reefs, while San Josecito exhibited extreme bleaching and coral mortality. For PC2, *Pocillopora* spp. and dead coral were positively associated, and *Porites* spp., turf, macroalgae, and CCA negatively associated. San Josecito had the highest positive score, while Barco Somero had the most negative, followed by Este Intermedio and Tina, indicating high turf cover at the latter sites. PC3 (16.9%) explained site variance based *Caulerpa* (positive), contrasting with coral cover and CCA.

### After bleaching (August to December 2024)

The PCA for August to December 2024 distinguished sites with high bleaching prevalence, turf algae, and coral loss from deeper sites, explaining 86% of the total variance among the first four PCs (PC1 32.6%, PC2 24.2%, PC3 15.3%, and PC4 13.9%). PC1 separated deeper sites (0.42), associated with higher coral cover, from sites with high bleaching prevalence (−0.32) and declining coral cover (−0.23). Ancla and Cueva clustered with deeper reefs, while San Josecito and Tina exhibited high bleaching and loss of coral cover. PC2 differentiated sites based on coral cover (−0.36) and CCA (−0.24), contrasting with turf algae (+0.40) and dead coral (−0.35). PC3 (15.3%) captured site differences in coral cover (−0.37) *versus* increasing turf algae dominance. PC4 (13.9%) reflected variation in macroalgae (−0.48) and total Other coral (−0.49), distinguishing sites with increased macroalgal presence from those with remaining coral dominance.

### Latest (January and February 2025)

The PCA for the latest data demonstrated a strong gradient distinguishing coral-dominated and algal-dominated reef sites. The first four PCs explained 86.74% of the total variance, with PC1 (34.5%) and PC2 (22.1%) as the dominant axes. PC1 primarily reflected a coral-to-algae gradient, with coral cover (−0.426) negatively associated and turf algae (0.347), CCA (0.279), and depth (0.366) positively associated. Barco Somero and Esquina aligned with coral-dominated reefs, while Barco Profundo and Cueva exhibited higher algal cover. PC2 (22.1%) captured variation in coral cover (0.279) and depth (0.304), further distinguishing site-level differences. Ancla and San Josecito showed strong divergence, between axes, but bleaching was not a major structuring variable in this period. San Josecito exhibited low coral cover (−0.43), high turf algae (0.35), consistent with post-bleaching impacts. Barco Somero and Esquina had relatively higher coral cover and Barco Profundo and Cueva were positioned towards algal dominance.

Comparing PCA outcomes across time periods, there was a shift in the dominant variables associated with structuring community composition. For example, coral-associated variables contributed strongly to PC1 during the bleaching period but were less influential in the most recent PCA, where turf algae had higher loadings. Turf algae became more strongly aligned with sites in ordination space post-bleaching, reflecting a continued shift in the principal structuring variables. Bleached coral initially played a key role in explaining community variation but was less strongly associated with PCA axes in later analyses, suggesting either coral mortality or stabilization of post-disturbance conditions. *Pavona* spp. showed more stable contributions to PC1 across all time periods, suggesting a consistent relationship with community composition, which may reflect resilience to disturbance and bleaching events.

### Ecological recovery feasibility index

**Figure 8:**
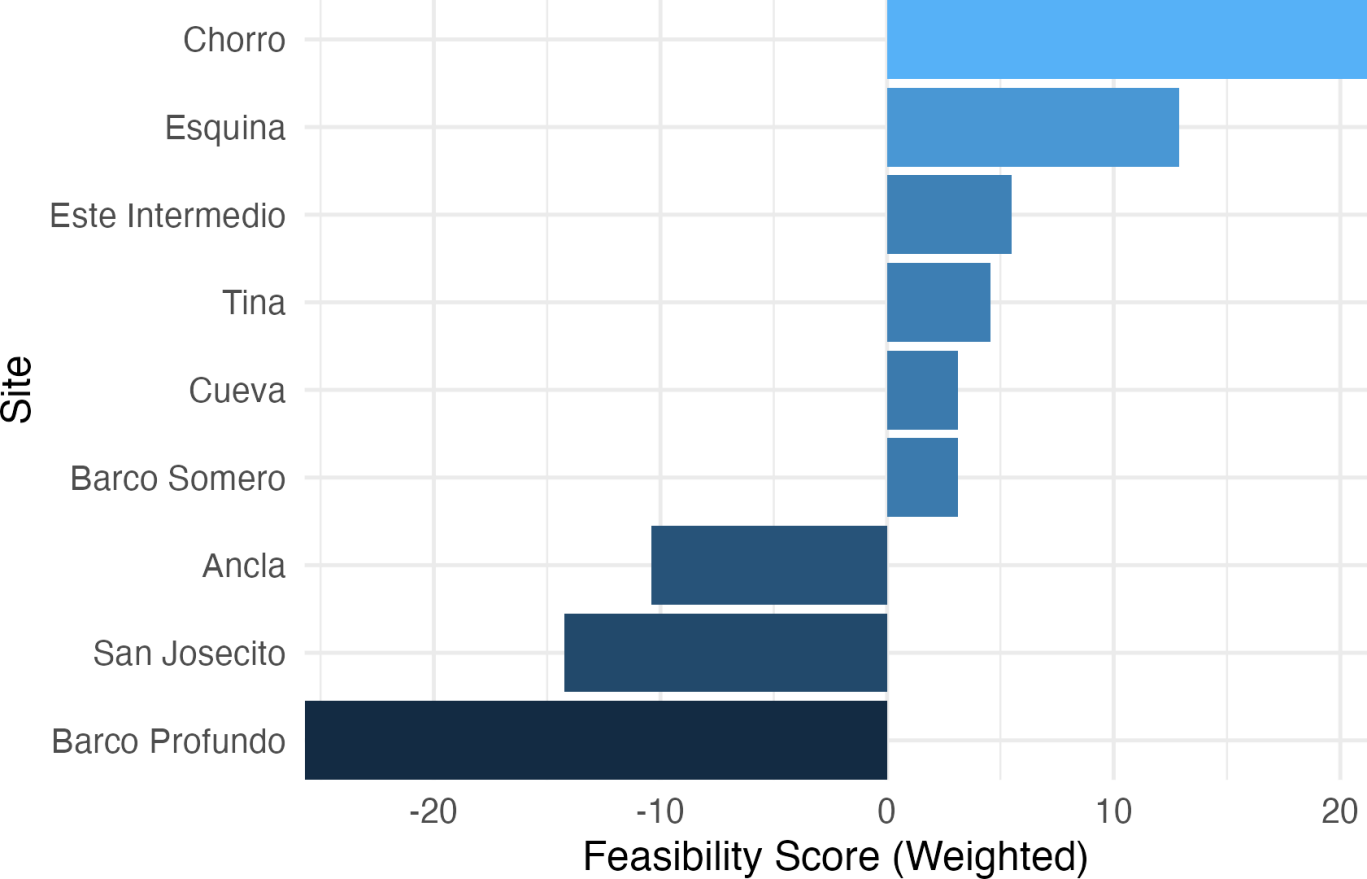
Ecological recovery feasibility index. A composite multi-metric index to synthesize complex ecological data into decision-support tools for prioritizing interventions.

The Ecological Recovery Feasibility Index (ERFI), which prioritizes coral cover and ecologically relevant PCA loadings (Fig. 8), revealed significant variation in reef restoration potential across sites. Chorro (21.18) and Esquina (12.89) exhibited the highest feasibility scores, indicating favorable conditions for coral recovery. Este Intermedio (5.49) and Tina (4.56) showed moderate restoration potential, suggesting a balance between coral persistence and algal encroachment. Cueva (3.15) and Barco Somero (3.11) also ranked positively but with lower scores, likely influenced by higher macroalgae and cyanobacteria cover. Conversely, Ancla (−10.41), San Josecito (−14.26), and Barco Profundo (−25.72) ranked lowest, suggesting degraded reef conditions that may hinder self-recovery. As expected given the index construction, sites with higher feasibility scores also exhibited greater coral cover and lower turf and cyanobacteria prevalence, whereas low-ranking sites were characterized by persistent algal dominance.

## Discussion

Climate-change driven ocean warming is more frequently pushing coral reefs beyond their thermal limits, causing extensive coral loss and ecosystem collapse (Armstrong McKay et al., 2022; Bland et al., 2018; Pearce-Kelly et al., 2025). The 2023–24 El Niño event destabilized Isla del Caño’s coral communities that have been reported as both biodiverse and resilient (Alvarado et al., 2020; Friedlander et al., 2022; Glynn et al., 2017; Romero-Torres et al., 2020). Thermal bleaching drove widespread coral mortality, with the highest recorded SST (31.2 °C) on top of four decades of temperature increase and reduced cooling, causing a shift from coral to turf algal dominance. Coral bleaching prevalence and associated declines were variable among species and site, with the highest loss at Ancla, Esquina, Tina at Isla del Caño and at San Josecito, on the north of the Osa Peninsula. The loss of coral cover and shift to algal dominance highlights an urgent need to develop a reactive, science-led and innovative coral reef recovery and restoration strategy among all local stakeholders (Kleypas et al., 2021).

### The 2023–24 El Niño surpassed historical SST at Isla del Caño

The 2023–24 El Niño marked the most extreme thermal event at Isla del Caño for 40 years, with SSTs reaching unprecedented levels, a triple-peaked DHW anomaly, and the lowest frequency of cool days. This surpasses prior El Niño events documented to affect Costa Rica’s ETP coral reefs since the 1980s (Alvarado et al., 2020; Cortés et al., 2010; Cortés & Jiménez, 2003; Guzmán et al., 1987; Jiménez et al., 2001) and signals a loss of climate variability that may have previously buffered reefs from thermal stress (Pearce-Kelly et al., 2025; Trew & Maclean, 2021). While extreme cooling events can cause coral die-off, such as in Costa Rica’s north Pacific (Palmer et al., 2022), cool periods reduce bleaching impact and aid reef recovery (Green et al., 2019). With the exception of the 1983 El Niño, Isla del Caño’s coral communities have previously escaped devastation from thermal anomalies (Alvarado et al., 2020; Jiménez et al., 2001), leading to the suggestion of local resilience (Alvarado et al., 2020; Friedlander et al., 2022; Guzmán & Cortés, 2007; Romero-Torres et al., 2020). However, retrospective analysis shows that locally the thermal events were not prolonged or extreme and were accompanied by cool days. Over the 40-year period from 1985 to 2025, SSTs at Isla del Caño have risen by 0.92 °C, slightly above the global average of 0.7 °C (Samset et al., 2023), and are consistent with global climate change and ENSO-induced variability (Intergovernmental Panel on Climate Change (IPCC), 2021). This change in thermal regime, with a warming trend decoupled from cool periods, suggests that thermal stress is likely to exceed the pace of natural coral reef recovery at Isla del Caño (Guzmán & Cortés, 2007), increasing the risk of a persistent shift to algal dominated reefs (Nyström et al., 2012; Pearce-Kelly et al., 2025).

### Coral to algal dominance

The 2023–24 El Niño triggered a rapid and severe shift from coral to algal dominance at Isla del Caño and the Osa Peninsula, signaling widespread reef degradation (Armstrong McKay et al., 2022; Bland et al., 2018; Darling et al., 2019; Nyström et al., 2012), a likely breach of a tipping point threshold (Pearce-Kelly et al., 2025) and the potential onset of an alternative stable state (Bruno et al., 2009; Crisp, Tebbett & Bellwood, 2022; Folke et al., 2004). Coral cover declined by over 40% and algal cover increased by more than 70%, though as reef sites were surveyed from the tail-end of the thermal anomaly (Reimer et al., 2024), it is likely that the coral loss documented is a gross underestimate. Furthermore, six of the nine sites had approximately 10% coral cover or less in the most recent benthic surveys (January and February 2025) commensurate with levels reported for ecosystem collapse, which is defined as the endpoint of ecosystem degradation (Bland et al., 2018). While the relatively short-term data does not confirm the stability of an alternative state, the observed ecological patterns are consistent with early indicators or such transitions (Fung, Seymour & Johnson, 2011). For example, the magnitude of change and potential for positive feedback loops, whereby persistent algal dominance suppresses coral growth and recruitment, suggest that recovery trajectories may be impaired (Eddy et al., 2021; Norström et al., 2009). The benthic community composition shift at Isla del Caño suggests a significant digression from its previously reported status as a resilient biodiversity hotspot (Cortés et al., 2010; Friedlander et al., 2022; Glynn et al., 2017a).

### Spatial and taxonomic trends of reef degradation

Variability in spatial and taxonomic trends highlights the influence of local conditions in buffering or promoting reef degradation in response to the thermal event (Winslow et al., 2024). The most substantial site-level changes were observed at San Josecito, Ancla, Barco Profundo, and Cueva, where significant coral loss, turf proliferation, and reductions in macroalgae and cyanobacteria occurred. Shallow reefs such as San Josecito and Tina, where *Pocillopora* spp. dominate (Cortés & Jiménez, 2003), exhibited early signs of degradation, with high bleaching prevalence and coral mortality, consistent with the genus’s thermal sensitivity (Marshall & Baird, 2000; Palmer, Bythell & Willis, 2010). In contrast, deeper reefs such as Ancla, Cueva, and Barco Profundo initially appeared more resistant, likely benefiting from cooler water suggestive of a depth-related bleaching refugia (Muir et al., 2017) and a higher prevalence of more thermally tolerant genera *Pavona* and *Psammocora* (Marshall & Baird, 2000; Palmer, 2018b; Palmer, Bythell & Willis, 2010). However, these deeper sites were not immune to degradation. *Pavona* spp., in particular, exhibited partial mortality, demonstrating breaches of damage thresholds, where accumulated stress exceed repair capacity (Palmer, 2018a). The resultant fragmentation inflated colony counts, creating a misleading perception of resistance. It is likely that the timings of the surveys, conducted after the peak bleaching, missed much of the acute mortality phase, particularly for *Pavona clavus*, which local observation indicate it had already severely declined. This highlights the need for continuous assessment to accurately track community changes and mortality timing (Obura et al., 2019).

In addition to thermal stress, site-specific degradation was compounded by synergistic local stressors. Cueva and Ancla were likely exposed to intense sedimentation, given their proximity to the November 2024 landslides, which likely contributed to coral declines by blocking light, altering microbial communities, and increasing the immune and energetic demands for sediment clearance (Sheridan et al., 2014). Additionally, nutrient influx from fine sediments likely promoted turf algae proliferation (Fourney & Figueiredo, 2017; Vermeij et al., 2010). These impacts were reflected in the SIMPER analysis, which identified turf algae as the primary contributor to benthic community change at Ancla, Cueva, and Barco Profundo.

Several sites, including Esquina, Este Intermedio, and Chorro, exhibited more gradual transitions, initially maintaining higher coral cover before progressively shifting toward turf dominance. Tina and Barco Somero also showed signs of decline, though degradation at Barco Somero was less severe, with turf algae not reaching the dominance levels observed at San Josecito or Barco Profundo. These patterns suggest that site-specific factors, such as initial community composition or local conditions, may have modulated the pace and extent of degradation (Norström et al., 2009).

Across all sites, only 12 coral taxa were recorded in coral reef surveys (2024 to 2025), a reduction from the 22 species historically reported at Isla del Caño (Cortés & Guzmán, 1998; Guzmán & Cortés, 2001). *Porites panamensis* was notably absent and may be locally extirpated, while *Gardineroseris planulata* was rare. Whilst our surveys grouped all *Pocillopora* spp. together, due to difficulties with identification to species level in the field (Johnston, Forsman & Toonen, 2018), their overall decline points to a loss of diversity that threatens long-term resilience (Folke et al., 2004; Nyström, Folke & Moberg, 2000). However, the persistence of some *Pocillopora* spp. colonies raises the possibility that these individuals may possess enhanced thermal tolerance, potentially through endosymbiont shuffling or the selective survival of more resilient species or genotypes (Palacio-Castro et al., 2023; Romero-Torres et al., 2020). Further impacting the system, crown-of-thorns starfish (*Acanthaster planci*) were observed preying on surviving coral, including *Porites lobata,* reducing reef recovery potential.

The cumulative effects of bleaching, sedimentation, and predation appear to have driven an ecological simplification at Isla del Caño and the north Osa Peninsula. Although bleaching was visually prevalent in 2023, its contribution to post-disturbance dissimilarity was low, and dead coral decreased at most sites, reflecting overgrowth of coral skeletons (Romanó De Orte et al., 2021). This suggests that the most significant coral loss occurred prior to the 2024 and 2025 surveys and that the true extent of mortality, and among-species variation, may be underestimated. The widespread decline in live coral cover reinforces this conclusion and indicates a substantial ecological shift, with many reefs converging towards a simplified, turf-dominated state that appears to be beginning to stabilize.

### Ecological recovery feasibility

Composite indices, which combine multiple ecological indicators into a single decision-support tool, are widely used in terrestrial and aquatic restoration ecology to synthesize complex ecological data into decision-support tools for prioritizing interventions and evaluating site-specific recovery potential (Orsi, Geneletti & Newton, 2011; Riedler et al., 2015). These frameworks provide transparent, replicable means to integrate multiple ecological indicators and have been instrumental in informing triage-based management in degraded ecosystems (Smith et al., 2023b). In coral reef restoration, the need for analogous tools has been emphasized to align restoration efforts with ecological baselines and resilience-based objectives (Hein et al., 2021; Shaver et al., 2020; Shaver et al., 2022). Responding to this gap, the ERFI introduced here offers a preliminary framework for evaluating the relative recovery potential of reef communities in Costa Rica. By integrating science-informed ecological trajectories, current benthic composition, and PCA-derived ecological gradients, the index can be used to facilitate triage-based restoration decisions at both local and regional scales (Kleypas et al., 2021; Obura et al., 2019). As with other composite indices, the resolution and accuracy of the ERFI will benefit from ongoing refinement and validation, particularly when applied to other reef systems (Smith et al., 2023b).

The application of the ERFI to Isla del Caño and northern Osa Peninsula reefs reveals substantial site-level variation in recovery potential. Critically, the index highlights sites such as San Josecito, Ancla, and Barco Profundo as having limited feasibility for natural recovery, underscoring the need for reactive restoration at these locations (Hein et al., 2021; Kleypas et al., 2021). In contrast, sites like Chorro and Esquina demonstrated relatively higher recovery scores yet remain potentially vulnerable to accelerating thermal anomalies.

These findings affirm that passive recovery alone is unlikely to restore ecological function at the most degraded reef sites. Proactive, site-specific interventions that are tailored to the ecological conditions and disturbance history of each reef are increasingly essential to sustaining biodiversity and ecosystem services in the face of compounding stressors (Alvarado et al., 2025; Pearce-Kelly et al., 2025).

### Management and restoration implications

The ERFI highlights that several reef sites in Isla del Caño and the northern Osa Peninsula, especially San Josecito, Ancla, and Barco Profundo, have crossed ecological thresholds, marked by steep coral loss and turf algae proliferation. These changes exceed permissible variability defined by SINAC’s ecological monitoring protocol, PRONAMEC, which calls for comprehensive impact mitigation but lacks clear ecological response strategies (Sistema Nacional de Áreas de Conservación (SINAC), 2016; Nacional de Áreas de Conservación (SINAC), 2021). The transition to turf dominance, particularly at high-use tourism sites such as Ancla, Cueva, and Barco Profundo, aligns with previous calls for more conservation efforts and sustainable use of the Osa Conservation Area (Alvarado et al., 2015), and underscores the need for urgent site-specific restoration and enhanced management (BIOMARCC-SINAC-GIZ, 2016; Naranjo-Arriola, 2021).

Coral gardening remains the most widely implemented restoration method globally, showing moderate success in boosting local coral cover, especially for fast-growing genera like *Pocillopora* and *Acropora* (Boström-Einarsson et al., 2020; Shaver et al., 2022; Suggett & Van Oppen, 2022). In Costa Rica, low-cost methods such as floating and benthic nurseries have enabled local-scale outplanting of both branching and massive corals, with promising growth (∼6–9 cm/year) and survival (60–90%) rates (Alvarado et al., 2025). However, these gains are fragile without addressing underlying stressors like turf algae overgrowth and thermal stress.

To stabilize benthic trajectories, ecological management must address both biotic and abiotic stressors. While manual macroalgae removal has proven effective in some contexts (Smith et al., 2023a), Isla del Caño’s primary issue is turf algae. Small-scale turf removal around coral colonies can improve coral health and offers an entry point for community-based stewardship (Cetz-Navarro et al., 2013). Meanwhile, strengthening top-down control through herbivore protection, especially of key grazing fish and invertebrates, can suppress algal dominance and support recovery (Alvarado, Cortés & Reyes-Bonilla, 2015; Mumby, Hastings & Edwards, 2007) though the negative impacts of bioerosion should be considered (Alvarado et al., 2016; Alvarado, Cortés & Reyes-Bonilla, 2015). Reinforcing the no-take status of Isla del Caño (ACOSA-TNC-UCI-ELAP, 2008) and expanding herbivore biomass monitoring should be prioritized. In parallel, addressing land-based sediment and nutrient inputs remains critical (Alvarado, Fernández & Cortés, 2009), especially amid ongoing coastal development (Hall, 2024).

In Costa Rica, where restoration is led by NGOs and community-based organizations within the SINAC framework (Alvarado et al., 2025; SINAC-GIZ, 2020), low-tech, scalable methods remain the most feasible. However, long-term reef resilience depends not only on managing present threats but also on maintaining key ecosystem processes, such as coral reproduction and larval dispersal (Kleypas et al., 2021; Romero-Torres et al., 2018; Shaver et al., 2022). Further investigation into these fundamental processes is needed within the region (Glynn et al., 2017b; Lequeux et al., 2018; Santiago-Valentín et al., 2019) to enable a strategic long-term restoration plan to be developed in line with Costa Rica’s national restoration protocol (SINAC-GIZ, 2020). Experimental strategies, such as larval enhancement, cryopreservation, microbiome manipulation, and assisted evolution, hold promise (Barshis et al., 2013; Bay et al., 2023; Suggett et al., 2024), though are currently limited to pilot stages in high-income settings due to cost and infrastructure demands (Bayraktarov et al., 2016; McLeod et al., 2022; Schmidt-Roach et al., 2025), with limited field-scale validation (Pearce-Kelly et al., 2025). Immune profiling, as proposed under the damage threshold hypothesis of coral susceptibility (Palmer, 2018a), offers a practical and comparatively low-cost strategy for understanding coral health dynamics and informing selective propagation. Rather than aiming to manipulate immunity, this approach focuses on identifying coral phenotypes with inherently higher baseline immune responses, such as antioxidants and phenoloxidase activity (Palmer, 2018b; Palmer, Mydlarz & Willis, 2008; Palmer, Bythell & Willis, 2010), which can be prioritized for nursery rearing and outplanting. This aligns with resilience-based restoration goals (Shaver et al., 2022; SINAC-GIZ, 2020) by enhancing the likelihood of survival under recurrent stress events, and may be particularly valuable in guiding cost-effective restoration in resource-limited settings (Klepac et al., 2024).

Reef recovery depends on coordinated governance and reducing local anthropogenic pressures, including from tourism (Alvarado et al., 2015). Isla del Caño, though a biological reserve (Sistema Nacional de Áreas de Conservación (SINAC), 2009), supports intensive marine tourism that benefits local economies and conservation (BIOMARCC-SINAC-GIZ, 2016; Naranjo-Arriola, 2021). Although reduced visitor numbers during COVID-19 did not influence the abundance of predatory fish (Valverde, Cambra & Espinoza, 2025), tourism visitation has led to coral breakage and potentially other negative impacts, such as from sunscreen-derived toxins (He et al., 2019; Lamb et al., 2014), at sites including Ancla, Barco Profundo, Cueva, and Esquina (Naranjo-Arriola, 2021). Management responses, such as rotating site access, redistributing visitor pressure, setting carrying capacities, and providing targeted guide and tourist training, should be urgently reviewed (ACOSA-TNC-UCI-ELAP, 2008; Alvarado et al., 2015). With reef health underpinning regional tourism and conservation funding, ongoing collaboration between SINAC, NGOs, and local tourism operators is essential to align reef use with long-term ecosystem resilience (Alvarado et al., 2025; Kleypas et al., 2021).

## Conclusions

Isla del Caño’s coral reef communities, historically considered climate-resilient, underwent a significant, El Niño-driven shift from coral to algal dominance in 2024, with coral cover declining by over 40% and turf algae increasing by more than 70%. Furthermore, the temporal scope of the coral reef monitoring was constrained to one year following the peaks of the 2023–24 El Niño event, suggesting that the scale of coral loss is underestimated. These results mark a critical ecological transition, suggesting that the resilience observed in past decades has been eroded under accelerating climate pressures.

By integrating long-term temperature trends, bleaching thresholds, and benthic community dynamics across nine sites, we provide a comprehensive and site-specific temporal assessment of reef degradation at Isla del Caño and one site on the north of the Osa Peninsula. The development of an Ecological Recovery Feasibility Index offers a novel, site-level tool for prioritizing reactive intervention. This simple tool enables triage-based conservation planning and can be applied to other regions and developed as additional information becomes available, such as coral recruitment rates, larval connectivity, more granular temperature data and herbivore density and diversity.

Passive recovery is unlikely to fully restore reef integrity at Isla del Caño and the Osa Peninsula, and urgent restoration with conservation actions are required to regain ecosystem function and mitigate further ecosystem collapse. Whilst global and national efforts to reduce climate impacts are ongoing, targeted, science-driven restoration aimed at increasing reef resilience is essential at Isla del Caño and the Osa Peninsula. Coral reef restoration activities must be developed and executed in collaboration with key stakeholders of this important biological reserve, including government, conservation organizations, scientists and dive tour operators.

##  Supplemental Information

10.7717/peerj.20088/supp-1 Supplemental Information 1 Annual frequency (days) of thermal events at Isla del Caño 8°42′59″N 83°53′06″W based on NOAA Coral Reef Watch SST data (1985–2025) Warm events are defined as days when SST exceeds the bleaching threshold (MMM + 0.5 °C). Cool events are defined as days when SST is below the monthly mean minus 0.5 °C.

10.7717/peerj.20088/supp-2 Supplemental Information 2 The landslides that occurred in November 2024 at Isla del Caño, photographed in January 2025 (A) the view from the beach of the extent of the landslide and amount of sediment that arrived at sea level, (B) the sediment plumes 2 months after the landslides and (C) the location of the landslides in relation to the survey sites at Isla del Caño (landslide location indicated with a star).

10.7717/peerj.20088/supp-3 Supplemental Information 3 Crown-of-thorns starfish predating on one of the few remaining *Pavona clavus* colonies at Isla del Caño

10.7717/peerj.20088/supp-4 Supplemental Information 4 LOESS-smoothed temporal trends for key benthic categories (coral, dead coral, turf, macroalgae, CCA, and cyanobacteria) across all survey sites Site-level changes over time and complements the SIMPER analysis by showing the raw cover trends that contribute to compositional shifts.

10.7717/peerj.20088/supp-5 Supplemental Information 5 Percent cover of coral and turf algae

10.7717/peerj.20088/supp-6 Supplemental Information 6 Boxplots of the mean temperature (HOBO temperature logger) at each reef site

10.7717/peerj.20088/supp-7 Supplemental Information 7 The proportion cover of each coral taxa at each site During Bleaching, After Bleaching and in the Latest data (January and February 2025)

10.7717/peerj.20088/supp-8 Supplemental Information 8 Site coordinates and depth ranges

10.7717/peerj.20088/supp-9 Supplemental Information 9 Sites and methods used for each survey since 2019-2025 in Caño Island, Costa Rica

10.7717/peerj.20088/supp-10 Supplemental Information 10 Worked example calculation of the Ecological Recovery Feasibility Index (ERFI)

10.7717/peerj.20088/supp-11 Supplemental Information 11 Summary of statistical models used in the study

10.7717/peerj.20088/supp-12 Supplemental Information 12 Presence/Absence of each coral species by site at Isla del Caño and Osa Peninsula

10.7717/peerj.20088/supp-13 Supplemental Information 13 Shannon Index full model Dunn’s *Post hoc* comparison

10.7717/peerj.20088/supp-14 Supplemental Information 14 Mean percentage coral cover for December 2024 to February 2025 (SIMPER)

10.7717/peerj.20088/supp-15 Supplemental Information 15 Tukey HSD results for site-wise mean monthly temperature comparisons

10.7717/peerj.20088/supp-16 Supplemental Information 16 Summary of regression estimates for coral cover trends

10.7717/peerj.20088/supp-17 Supplemental Information 17 Mean benthic cover (% SD) at most recent survey by site

10.7717/peerj.20088/supp-18 Supplemental Information 18 Summary of PCA outputs

10.7717/peerj.20088/supp-19 Supplemental Information 19 Top PCA loadings summary table

10.7717/peerj.20088/supp-20 Supplemental Information 20 Water temperature data from deployed loggers

10.7717/peerj.20088/supp-21 Supplemental Information 21 The survey data

10.7717/peerj.20088/supp-22 Supplemental Information 22 Markdown file for coral declines at Isla del Cano

10.7717/peerj.20088/supp-23 Supplemental Information 23 Local temperature analysis data code

10.7717/peerj.20088/supp-24 Supplemental Information 24 NOAA CRW data for Isla del Cano

10.7717/peerj.20088/supp-25 Supplemental Information 25 Markdown file for NOAA Coral Reef Watch data analysis

## Additional Information and Declarations

### Data Availability

The following information was supplied regarding data availability: The raw data is available in the Supplemental Files.

## References

[ref-1] ACOSA-TNC-UCI-ELAP (2008). Executive summary of the Caño Island Biological Reserve Management Plan. https://biocorredores.org/corredoresbiologicos/sites/default/files/docs/SerieTecnica17_CaracterizacionBuceoAMP.pdf.

[ref-2] Alvarado JJ, Beita-Jiménez A, Mena S, Fernández-García C, Guzmán-Mora AG (2015). Osa Conservation Area (Costa Rica) coral ecosystems: structure and conservation needs. Revista de Biología Tropical.

[ref-3] Alvarado JJ, Cortés J, Guzman H, Reyes-Bonilla H (2016). Bioerosion by the sea urchin *Diadema mexicanum* along Eastern Tropical Pacific coral reefs. Marine Ecology.

[ref-4] Alvarado JJ, Cortés J, Reyes-Bonilla H (2015). Reconstruction of *Diadema mexicanum* bioerosion impact on three Costa Rican Pacific coral reefs. Revista de Biología Tropical.

[ref-5] Alvarado JJ, Evans K, Kleypas J, Marin-Moraga JA, Mendez-Venegas M, Perez-Reyes C, Sandoval M, Solano MJ, Villalobos Cubero T (2025). Coral reefs restoration initiatives in Costa Rica: ten years building hope. Revista de Biología Tropical.

[ref-6] Alvarado JJ, Fernández C, Cortés J (2009). Water quality conditions on coral reefs at the Marino Ballena National Park, Pacific Costa Rica. Bulletin of Marine Science.

[ref-7] Alvarado JJ, Sánchez-Noguera C, Arias-Godínez G, Araya T, Fernández-García C, Guzmán AG (2020). Impact of El Niño 2015–2016 on the coral reefs of the Pacific of Costa Rica: the potential role of marine protection. Revista de Biología Tropical.

[ref-8] Armstrong McKay DI, Staal A, Abrams JF, Winkelmann R, Sakschewski B, Loriani S, Fetzer I, Cornell SE, Rockström J, Lenton TM (2022). Exceeding 1.5 °C global warming could trigger multiple climate tipping points. Science.

[ref-9] Barlow J, França F, Gardner TA, Hicks CC, Lennox GD, Berenguer E, Castello L, Economo EP, Ferreira J, Guénard B, Gontijo Leal C, Isaac V, Lees AC, Parr CL, Wilson SK, Young PJ, Graham NAJ (2018). The future of hyperdiverse tropical ecosystems. Nature.

[ref-10] Barshis DJ, Ladner JT, Oliver TA, Seneca FO, Traylor-Knowles N, Palumbi SR (2013). Genomic basis for coral resilience to climate change. Proceedings of the National Academy of Sciences of the United States of America.

[ref-11] Bay LK, Gilmour J, Muir B, Hardisty PE (2023). Management approaches to conserve Australia’s marine ecosystem under climate change. Science.

[ref-12] Bayraktarov E, Saunders MI, Abdullah S, Mills M, Beher J, Possingham HP, Mumby PJ, Lovelock CE (2016). The cost and feasibility of marine coastal restoration. Ecological Applications.

[ref-13] BIOMARCC-SINAC-GIZ (2016). Characterization of recreational diving activities in Costa Rica’s marine protected areas. https://biocorredores.org/corredoresbiologicos/sites/default/files/docs/SerieTecnica17_CaracterizacionBuceoAMP.pdf.

[ref-14] Bland LM, Rowland JA, Regan TJ, Keith DA, Murray NJ, Lester RE, Linn M, Rodríguez JP, Nicholson E (2018). Developing a standardized definition of ecosystem collapse for risk assessment. Frontiers in Ecology and the Environment.

[ref-15] Boström-Einarsson L, Babcock RC, Bayraktarov E, Ceccarelli D, Cook N, Ferse SCA, Hancock B, Harrison P, Hein M, Shaver E, Smith A, Suggett D, Stewart-Sinclair PJ, Vardi T, McLeod IM (2020). Coral restoration—a systematic review of current methods, successes, failures and future directions. PLOS ONE.

[ref-16] Bruno JF, Sweatman H, Precht WF, Selig ER, Schutte VGW (2009). Assessing evidence of phase shifts from coral to macroalgal dominance on coral reefs. Ecology.

[ref-17] Bürkner P-C (2017). brms: an *R* package for bayesian multilevel models using *Stan*. Journal of Statistical Software.

[ref-18] Carpenter B, Gelman A, Hoffman MD, Lee D, Goodrich B, Betancourt M, Brubaker M, Guo J, Li P, Riddell A (2017). Stan: a probabilistic programming language. Journal of Statistical Software.

[ref-19] Cetz-Navarro NP, Espinoza-Avalos J, Hernández-Arana HA, Carricart-Ganivet JP (2013). Biological responses of the coral montastraea annularis to the removal of filamentous turf algae. PLOS ONE.

[ref-20] Claar DC, Szostek L, McDevitt-Irwin JM, Schanze JJ, Baum JK (2018). Global patterns and impacts of El Niño events on coral reefs: a meta-analysis. PLOS ONE.

[ref-21] Cortés J, Guzmán HM (1998). Organismos de los arrecifes coralinos de Costa Rica: Descripción, distribución geográfica e historia natural de los corales zooxantelados (Anthozoa: ScIeractinia) del Pacífico. Revista de Biología Tropical.

[ref-22] Cortés J, Jiménez C, Cortés J (2003). Corals and coral reefs of the Pacific of Costa Rica: history, research and status. Latin American coral reefs.

[ref-23] Cortés J, Jiménez C, Fonseca AC, Alvarado JJ (2010). Status and conservation of coral reefs in Costa Rica. Revista de Biología Tropical.

[ref-24] Crisp SK, Tebbett SB, Bellwood DR (2022). A critical evaluation of benthic phase shift studies on coral reefs. Marine Environmental Research.

[ref-25] Darling ES, McClanahan TR, Maina J, Gurney GG, Graham NAJ, Januchowski-Hartley F, Cinner JE, Mora C, Hicks CC, Maire E, Puotinen M, Skirving WJ, Adjeroud M, Ahmadia G, Arthur R, Bauman AG, Beger M, Berumen ML, Bigot L, Bouwmeester J, Brenier A, Bridge TCL, Brown E, Campbell SJ, Cannon S, Cauvin B, Chen CA, Claudet J, Denis V, Donner SD, Estradivari, Fadli N, Feary DA, Fenner D, Fox H, Franklin EC, Friedlander A, Gilmour J, Goiran C, Guest J, Hobbs J-PA, Hoey AS, Houk P, Johnson S, Jupiter SD, Kayal M, Kuo C-Y, Lamb J, Lee MAC, Low J, Muthiga N, Muttaqin E, Nand Y, Nash KL, Nedlic O, Pandolfi JM, Pardede S, Patankar V, Penin L, Ribas-Deulofeu L, Richards Z, Roberts TE, Rodgers KS, Safuan CDM, Sala E, Shedrawi G, Sin TM, Smallhorn-West P, Smith JE, Sommer B, Steinberg PD, Sutthacheep M, Tan CHJ, Williams GJ, Wilson S, Yeemin T, Bruno JF, Fortin M-J, Krkosek M, Mouillot D (2019). Social–environmental drivers inform strategic management of coral reefs in the Anthropocene. Nature Ecology & Evolution.

[ref-26] Dudley N (2008). Guidelines for applying protected area management categories. IUCN.en. https://portals.iucn.org/library/sites/library/files/documents/pag-021.pdf.

[ref-27] Eakin CM, Sweatman HPA, Brainard RE (2019). The 2014–2017 global-scale coral bleaching event: insights and impacts. Coral Reefs.

[ref-28] Eddy TD, Lam VWY, Reygondeau G, Cisneros-Montemayor AM, Greer K, Palomares MLD, Bruno JF, Ota Y, Cheung WWL (2021). Global decline in capacity of coral reefs to provide ecosystem services. One Earth.

[ref-29] Edwards A, Guest J, Humanes A (2024). Rehabilitating coral reefs in the Anthropocene. Current Biology.

[ref-30] Fernández C (2007). Propagation of the algae Caulerpa sertularioides in Culebra Bay, Gulf of Papagayo. Master’s Thesis.

[ref-31] Folke C, Carpenter S, Walker B, Scheffer M, Elmqvist T, Gunderson L, Holling CS (2004). Regime shifts, resilience, and biodiversity in ecosystem management. Annual Review of Ecology, Evolution, and Systematics.

[ref-32] Ford AK, Bejarano S, Nugues MM, Visser PM, Albert S, Ferse SCA (2018). Reefs under Siege—the rise, putative drivers, and consequences of benthic cyanobacterial mats. Frontiers in Marine Science.

[ref-33] Fourney F, Figueiredo J (2017). Additive negative effects of anthropogenic sedimentation and warming on the survival of coral recruits. Scientific Reports.

[ref-34] Friedlander AM, Ballesteros E, Breedy O, Naranjo-Elizondo B, Hernández N, Salinas-de León P, Sala E, Cortés J (2022). Nearshore marine biodiversity of Osa Peninsula, Costa Rica: where the ocean meets the rainforest. PLOS ONE.

[ref-35] Fung T, Seymour RM, Johnson CR (2011). Alternative stable states and phase shifts in coral reefs under anthropogenic stress. Ecology.

[ref-36] Glynn PW, Alvarado JJ, Banks S, Cortés J, Feingold JS, Jiménez C, Maragos JE, Martínez P, Maté JL, Moanga DA, Navarrete S, Reyes-Bonilla H, Riegl B, Rivera F, Vargas-Ángel B, Wieters EA, Zapata FA, Glynn PW, Manzello DP, Enochs IC (2017a). Eastern Pacific Coral reef provinces, coral community structure and composition: an overview. Coral reefs of the Eastern Tropical Pacific.

[ref-37] Glynn PW, Colley SB, Carpizo-Ituarte E, Richmond RH, Glynn PW, Manzello DP, Enochs IC (2017b). Coral reproduction in the Eastern Pacific. Coral reefs of the Eastern Tropical Pacific.

[ref-38] Glynn PW, Mones AB, Podestá GP, Colbert A, Colgan MW, Glynn PW, Manzello DP, Enochs IC (2017). El Niño-Southern oscillation: effects on Eastern Pacific coral reefs and associated biota. Coral reefs of the Eastern Tropical Pacific.

[ref-39] Goergen EA, Lustic C, Levy J, Johnson ME, Griffin S, Moulding AL, Ross A (2025). Guide to coral reef restoration: optimizing efficiency and scale for *in situ* nurseries and outplanting.

[ref-40] Green RH, Lowe RJ, Buckley ML, Foster T, Gilmour JP (2019). Physical mechanisms influencing localized patterns of temperature variability and coral bleaching within a system of reef atolls. Coral Reefs.

[ref-41] Guzmán HM (1991). Restoration of coral reefs in pacific costa rica. Conservation Biology.

[ref-42] Guzmán HM, Cortés J (2001). Changes in reef community structure after fifteen years of natural disturbances in the Eastern Pacific (Costa Rica). Bulletin of Marine Science.

[ref-43] Guzmán HM, Cortés J (2007). Reef recovery 20 years after the 1982–1983 El Niño massive mortality. Marine Biology.

[ref-44] Guzmán HM, Cortés J, Richmond RH, Glynn PW (1987). Effects of the 1982/83 El Niño Southern Oscillation on the coral reefs of Caño Island. Costa Rica.

[ref-45] Hall E (2024). Dominical’s water crisis: the shocking truth behind the illegal development project. The Tico Times.

[ref-46] He T, Tsui MMP, Tan CJ, Ma CY, Yiu SKF, Wang LH, Chen TH, Fan TY, Lam PKS, Murphy MB (2019). Toxicological effects of two organic ultraviolet filters and a related commercial sunscreen product in adult corals. Environmental Pollution.

[ref-47] Hein MY, Vardi T, Shaver EC, Pioch S, Boström-Einarsson L, Ahmed M, Grimsditch G, McLeod IM (2021). Perspectives on the use of coral reef restoration as a strategy to support and improve reef ecosystem services. Frontiers in Marine Science.

[ref-48] Heron SF, Liu G, Eakin CM, Skirving W, Muller-Karger FE, Vera-Rodriguea M, De La Cour J, Burgess TFR, Strong AE, Geiger EF, Guild LS, Lynds S (2015). Climatology development for NOAA coral reef watch’s 5-km product suite. NOAA Technical Report; No. Volume 145.

[ref-49] Heron SF, Maynard JA, Van Hooidonk R, Eakin CM (2016). Warming trends and bleaching stress of the world’s coral reefs 1985–2012. Scientific Reports.

[ref-50] Intergovernmental Panel on Climate Change (IPCC) (2021). Climate change 2021: The physical science basis: working group I contribution to the sixth assessment report of the intergovernmental panel on climate change.

[ref-51] Intergovernmental Panel on Climate Change (IPCC) (2022a). Climate change 2022: Impacts, adaptation and vulnerability: working group II contribution to the sixth assessment report of the intergovernmental panel on climate change.

[ref-52] Masson-Delmotte V, Zhai P, Pörtner HO, Roberts D, Skea J, Shukla PR, Pirani A, Moufouma-Okia W, Péan C, Pidcock R, Connors S, Matthews JBR, Chen Y, Zhou X, Gomis MI, Lonnoy E, Maycock T, Tignor M, Waterfield T, Intergovernmental Panel on Climate Change (IPCC) (2022b). Global warming of 1.5 °C. An IPCC special report on the impacts of global warming of 1.5 °C above pre-industrial levels and related global greenhouse gas emission pathways, in the context of strengthening the global response to the threat of climate change, sustainable development, and efforts to eradicate poverty.

[ref-53] Brondizio E, Diaz S, Settele J, Ngo HT, IPBES (2019). Zenodo.

[ref-54] Jiménez C, Cortés J, León A, Ruíz E (2001). Coral bleaching and mortality associated with the 1997–98 El Niño in an upwelling environment in the eastern Pacific (Gulf of Papagayo, Costa Rica). Bulletin of Marine Science.

[ref-55] Johnson JV, Dick JTA, Pincheira-Donoso D (2022). Local anthropogenic stress does not exacerbate coral bleaching under global climate change. Global Ecology and Biogeography.

[ref-56] Johnston EC, Forsman ZH, Toonen RJ (2018). A simple molecular technique for distinguishing species reveals frequent misidentification of Hawaiian corals in the genus *Pocillopora*. PeerJ.

[ref-57] Klein SG, Roch C, Duarte CM (2024). Systematic review of the uncertainty of coral reef futures under climate change. Nature Communications.

[ref-58] Klepac CN, Petrik CG, Karabelas E, Owens J, Hall ER, Muller EM (2024). Assessing acute thermal assays as a rapid screening tool for coral restoration. Scientific Reports.

[ref-59] Kleypas J, Allemand D, Anthony K, Baker AC, Beck MW, Hale LZ, Hilmi N, Hoegh-Guldberg O, Hughes T, Kaufman L, Kayanne H, Magnan AK, Mcleod E, Mumby P, Palumbi S, Richmond RH, Rinkevich B, Steneck RS, Voolstra CR, Wachenfeld D, Gattuso J-P (2021). Designing a blueprint for coral reef survival. Biological Conservation.

[ref-60] Lachs L, Donner SD, Mumby PJ, Bythell JC, Humanes A, East HK, Guest JR (2023). Emergent increase in coral thermal tolerance reduces mass bleaching under climate change. Nature Communications.

[ref-61] Lamb JB, True JD, Piromvaragorn S, Willis BL (2014). Scuba diving damage and intensity of tourist activities increases coral disease prevalence. Biological Conservation.

[ref-62] Lequeux BD, Ahumada-Sempoal M-A, López-Pérez A, Reyes-Hernández C (2018). Coral connectivity between equatorial eastern Pacific marine protected areas: a biophysical modeling approach. PLOS ONE.

[ref-63] Liu G, Eakin CM, Chen M, Kumar A, De La Cour JL, Heron SF, Geiger EF, Skirving WJ, Tirak KV, Strong AE (2018). Predicting heat stress to inform reef management: NOAA coral reef watch’s 4-month coral bleaching outlook. Frontiers in Marine Science.

[ref-64] Liu G, Strong AE, Skirving W (2003). Remote sensing of sea surface temperatures during 2002 Barrier Reef coral bleaching. Eos, Transactions American Geophysical Union.

[ref-65] Marshall PA, Baird AH (2000). Bleaching of corals on the great barrier reef: differential susceptibilities among taxa. Coral Reefs.

[ref-66] McLeod IM, Hein MY, Babcock R, Bay L, Bourne DG, Cook N, Doropoulos C, Gibbs M, Harrison P, Lockie S, Van Oppen MJH, Mattocks N, Page CA, Randall CJ, Smith A, Smith HA, Suggett DJ, Taylor B, Vella KJ, Wachenfeld D, Boström-Einarsson L (2022). Coral restoration and adaptation in Australia: the first five years. PLOS ONE.

[ref-67] Muir PR, Marshall PA, Abdulla A, Aguirre JD (2017). Species identity and depth predict bleaching severity in reef-building corals: shall the deep inherit the reef?. Proceedings of the Royal Society B: Biological Sciences.

[ref-68] Mumby PJ, Hastings A, Edwards HJ (2007). Thresholds and the resilience of Caribbean coral reefs. Nature.

[ref-69] Naranjo-Arriola A (2021). Tourist carrying capacity as a sustainability management tool for coral reefs in Caño Island Biological Reserve, Costa Rica. Ocean & Coastal Management.

[ref-70] Norström A, Nyström M, Lokrantz J, Folke C (2009). Alternative states on coral reefs: beyond coral–macroalgal phase shifts. Marine Ecology Progress Series.

[ref-71] Nyström M, Folke C, Moberg F (2000). Coral reef disturbance and resilience in a human-dominated environment. Trends in Ecology & Evolution.

[ref-72] Nyström M, Graham NAJ, Lokrantz J, Norström AV (2008). Capturing the cornerstones of coral reef resilience: linking theory to practice. Coral Reefs.

[ref-73] Nyström M, Norström AV, Blenckner T, De La Torre-Castro M, Eklöf JS, Folke C, Österblom H, Steneck RS, Thyresson M, Troell M (2012). Confronting feedbacks of degraded marine ecosystems. Ecosystems.

[ref-74] Obura DO, Aeby G, Amornthammarong N, Appeltans W, Bax N, Bishop J, Brainard RE, Chan S, Fletcher P, Gordon TAC, Gramer L, Gudka M, Halas J, Hendee J, Hodgson G, Huang D, Jankulak M, Jones A, Kimura T, Levy J, Miloslavich P, Chou LM, Karger F, Osuka K, Samoilys M, Simpson SD, Tun K, Wongbusarakum S (2019). Coral reef monitoring, reef assessment technologies, and ecosystem-based management. Frontiers in Marine Science.

[ref-75] Orsi F, Geneletti D, Newton AC (2011). Towards a common set of criteria and indicators to identify forest restoration priorities: an expert panel-based approach. Ecological Indicators.

[ref-76] Palacio-Castro AM, Smith TB, Brandtneris V, Snyder GA, Van Hooidonk R, Maté JL, Manzello D, Glynn PW, Fong P, Baker AC (2023). Increased dominance of heat-tolerant symbionts creates resilient coral reefs in near-term ocean warming. Proceedings of the National Academy of Sciences of the United States of America.

[ref-77] Palmer CV (2018a). Immunity and the coral crisis. Communications Biology.

[ref-78] Palmer CV (2018b). Warmer water affects immunity of a tolerant reef coral. Frontiers in Marine Science.

[ref-79] Palmer CV, Bythell JC, Willis BL (2010). Levels of immunity parameters underpin bleaching and disease susceptibility of reef corals. The FASEB Journal.

[ref-80] Palmer CV, Jimenez C, Bassey G, Ruiz E, Villalobos Cubero T, Chavarria Diaz MM, Harrison XA, Puschendorf R (2022). Cold water and harmful algal blooms linked to coral reef collapse in the Eastern Tropical Pacific. PeerJ.

[ref-81] Palmer CV, Mydlarz LD, Willis BL (2008). Evidence of an inflammatory-like response in non-normally pigmented tissues of two scleractinian corals. Proceedings of the Royal Society B: Biological Sciences.

[ref-82] Pearce-Kelly P, Altieri AH, Bruno JF, Cornwall CE, McField M, Muñiz Castillo AI, Rocha J, Setter RO, Sheppard C, Roman-Cuesta RM, Yesson C (2025). Considerations for determining warm-water coral reef tipping points. Earth System Dynamics.

[ref-83] R Core Team (2021). https://www.R-project.org/.

[ref-84] Reimer JD, Peixoto RS, Davies SW, Traylor-Knowles N, Short ML, Cabral-Tena RA, Burt JA, Pessoa I, Banaszak AT, Winters RS, Moore T, Schoepf V, Kaullysing D, Calderon-Aguilera LE, Wörheide G, Harding S, Munbodhe V, Mayfield A, Ainsworth T, Vardi T, Eakin CM, Pratchett MS, Voolstra CR (2024). The fourth global coral bleaching event: where do we go from here?. Coral Reefs.

[ref-85] Riedler B, Pernkopf L, Strasser T, Lang S, Smith G (2015). A composite indicator for assessing habitat quality of riparian forests derived from Earth observation data. International Journal of Applied Earth Observation and Geoinformation.

[ref-86] Romanó De Orte M, Koweek DA, Cyronak T, Takeshita Y, Griffin A, Wolfe K, Szmant A, Whitehead R, Albright R, Caldeira K (2021). Unexpected role of communities colonizing dead coral substrate in the calcification of coral reefs. Limnology and Oceanography.

[ref-87] Romero-Torres M, Acosta A, Palacio-Castro AM, Treml EA, Zapata FA, Paz-García DA, Porter JW (2020). Coral reef resilience to thermal stress in the Eastern Tropical Pacific. Global Change Biology.

[ref-88] Romero-Torres M, Treml EA, Acosta A, Paz-García DA (2018). The eastern tropical pacific coral population connectivity and the role of the eastern pacific barrier. Scientific Reports.

[ref-89] Salas E, Sánchez-Godínez C, Montero-Cordero A (2016). Marine fishes of Caño island biological reserve: reef fish community structure and updated list for the coastal fish. Revista de Biología Tropical.

[ref-90] Samset BH, Zhou C, Fuglestvedt JS, Lund MT, Marotzke J, Zelinka MD (2023). Steady global surface warming from 1973 to 2022 but increased warming rate after 1990. Communications Earth & Environment.

[ref-91] Santiago-Valentín JD, Rodríguez-Troncoso AP, Bautista-Guerrero E, López-Pérez A, Cupul-Magaña AL (2019). Successful sexual reproduction of the scleractinian coral *Porites panamensis*: evidence of planktonic larvae and recruitment. Invertebrate Biology.

[ref-92] Schmidt-Roach S, Knorr T, Roch C, Klaus R, Klepac C, Klein SG, Duarte CM (2025). Cost-efficiency and effectiveness of coral restoration pathways. Restoration Ecology.

[ref-93] Shaver EC, Courtney CA, West JM, Maynard J, Hein M, Wagner C, Philibotte J, MacGowan P, McLeod I, Boström-Einarsson L, Bucchianeri K, Johnston L, Koss J (2020). A manager’s guide to coral reef restoration planning and design (NOAA Technical Memorandum CRCP 36.

[ref-94] Shaver EC, McLeod E, Hein MY, Palumbi SR, Quigley K, Vardi T, Mumby PJ, Smith D, Montoya-Maya P, Muller EM, Banaszak AT, McLeod IM, Wachenfeld D (2022). A roadmap to integrating resilience into the practice of coral reef restoration. Global Change Biology.

[ref-95] Sheridan C, Grosjean PH, Leblud J, Palmer CV, Kushmaro A, Eeckhaut I (2014). Sedimentation rapidly induces an immune response and depletes energy stores in a hard coral. Coral Reefs.

[ref-96] Sistema Nacional de Áreas de Conservación (SINAC) (2009). CRANES II: Proposed territorial planning for biodiversity conservation in Costa Rica. Analysis of gaps in the representativeness and integrity of biodiversity in marine and coastal systems.

[ref-97] Sistema Nacional de Áreas de Conservación (SINAC) (2016). Implementation of protocols: monitoring of coral formations, and evaluation and selection of dive sites in the marine sector of the Osa Conservation Area: Isla del Caño Biological Reserve and Marino Ballena National Park.

[ref-98] Sistema Nacional de Áreas de Conservación (SINAC) (2021). National Protocol for Ecological Monitoring (PRONAMEC) of Rocky Reefs.

[ref-99] Sistema Nacional de Áreas de Conservación (SINAC) (2023). Coral formation monitoring report in Marino Ballena National Park and the Isla del Caño Biological Reserve.

[ref-100] SINAC-GIZ (2020). Protocol for the restoration of reefs and coral communities in Costa Rica.

[ref-101] SINAC-UNA (2021). National Protocol for Ecological Monitoring (PRONAMEC) of Rocky Reefs.

[ref-102] Sing Wong A, Vrontos S, Taylor ML (2022). An assessment of people living by coral reefs over space and time. Global Change Biology.

[ref-103] Skirving W, Marsh B, De La Cour J, Liu G, Harris A, Maturi E, Geiger E, Eakin CM (2020). CoralTemp and the coral reef watch coral bleaching heat stress product suite version 3.1. Remote Sensing.

[ref-104] Smith HA, Fulton SE, McLeod IM, Page CA, Bourne DG (2023a). Sea-weeding: manual removal of macroalgae facilitates rapid coral recovery. Journal of Applied Ecology.

[ref-105] Smith LM, Reschke EM, Bousquin JJ, Cheskiewicz LP, Ilias N, Kevin Summers J, Harvey JE (2023b). Methods for a composite ecological suitability measure to inform cumulative restoration assessments in Gulf of Mexico estuaries. Ecological Indicators.

[ref-106] Su J, Yin B, Chen L, Gasparatos A (2022). Priority areas for mixed-species mangrove restoration: the suitable species in the right sites. Environmental Research Letters.

[ref-107] Suggett DJ, Goergen EA, Fraser M, Hein MY, Hoot W, McLeod I, Montoya-Maya PH, Moore T, Ross AM, Vardi T (2025). A user’s guide to coral reef restoration terminologies. Coral Reefs.

[ref-108] Suggett DJ, Guest J, Camp EF, Edwards A, Goergen L, Hein M, Humanes A, Levy JS, Montoya-Maya PH, Smith DJ, Vardi T, Winters RS, Moore T (2024). Restoration as a meaningful aid to ecological recovery of coral reefs. Npj Ocean Sustainability.

[ref-109] Suggett DJ, Van Oppen MJH (2022). Horizon scan of rapidly advancing coral restoration approaches for 21st century reef management. Emerging Topics in Life Sciences.

[ref-110] Tebben J, Motti CA, Siboni N, Tapiolas DM, Negri AP, Schupp PJ, Kitamura M, Hatta M, Steinberg PD, Harder T (2015). Chemical mediation of coral larval settlement by crustose coralline algae. Scientific Reports.

[ref-111] Trew BT, Maclean IMD (2021). Vulnerability of global biodiversity hotspots to climate change. Global Ecology and Biogeography.

[ref-112] Valverde C, Cambra M, Espinoza M (2025). No effect of the COVID-19 lockdown on predatory fish abundance in the Caño Island Biological Reserve, Eastern Tropical Pacific Ocean. Regional Studies in Marine Science.

[ref-113] Van Oppen MJH, Gates RD, Blackall LL, Cantin N, Chakravarti LJ, Chan WY, Cormick C, Crean A, Damjanovic K, Epstein H, Harrison PL, Jones TA, Miller M, Pears RJ, Peplow LM, Raftos DA, Schaffelke B, Stewart K, Torda G, Wachenfeld D, Weeks AR (2017). Shifting paradigms in restoration of the world’s coral reefs. Global Change Biology.

[ref-114] Vermeij MJA, Van Moorselaar I, Engelhard S, Hörnlein C, Vonk SM, Visser PM (2010). The effects of nutrient enrichment and herbivore abundance on the ability of turf algae to overgrow coral in the caribbean. PLOS ONE.

[ref-115] Virgen-Urcelay A, Donner SD (2023). Increase in the extent of mass coral bleaching over the past half-century, based on an updated global database. PLOS ONE.

[ref-116] Wilkinson CR (1999). Global and local threats to coral reef functioning and existence: review and predictions. Marine and Freshwater Research.

[ref-117] Winslow EM, Speare KE, Adam TC, Burkepile DE, Hench JL, Lenihan HS (2024). Corals survive severe bleaching event in refuges related to taxa, colony size, and water depth. Scientific Reports.

