# Supplementary figures and images for: El Niño-driven phase shift to algal dominance on Isla del Caño’s coral reefs: implications for urgent restoration
Source: PeerJ. 2025 Nov 20;13:e20088. doi: 10.7717/peerj.20088 (PMC12640635; doi:10.7717/peerj.20088)

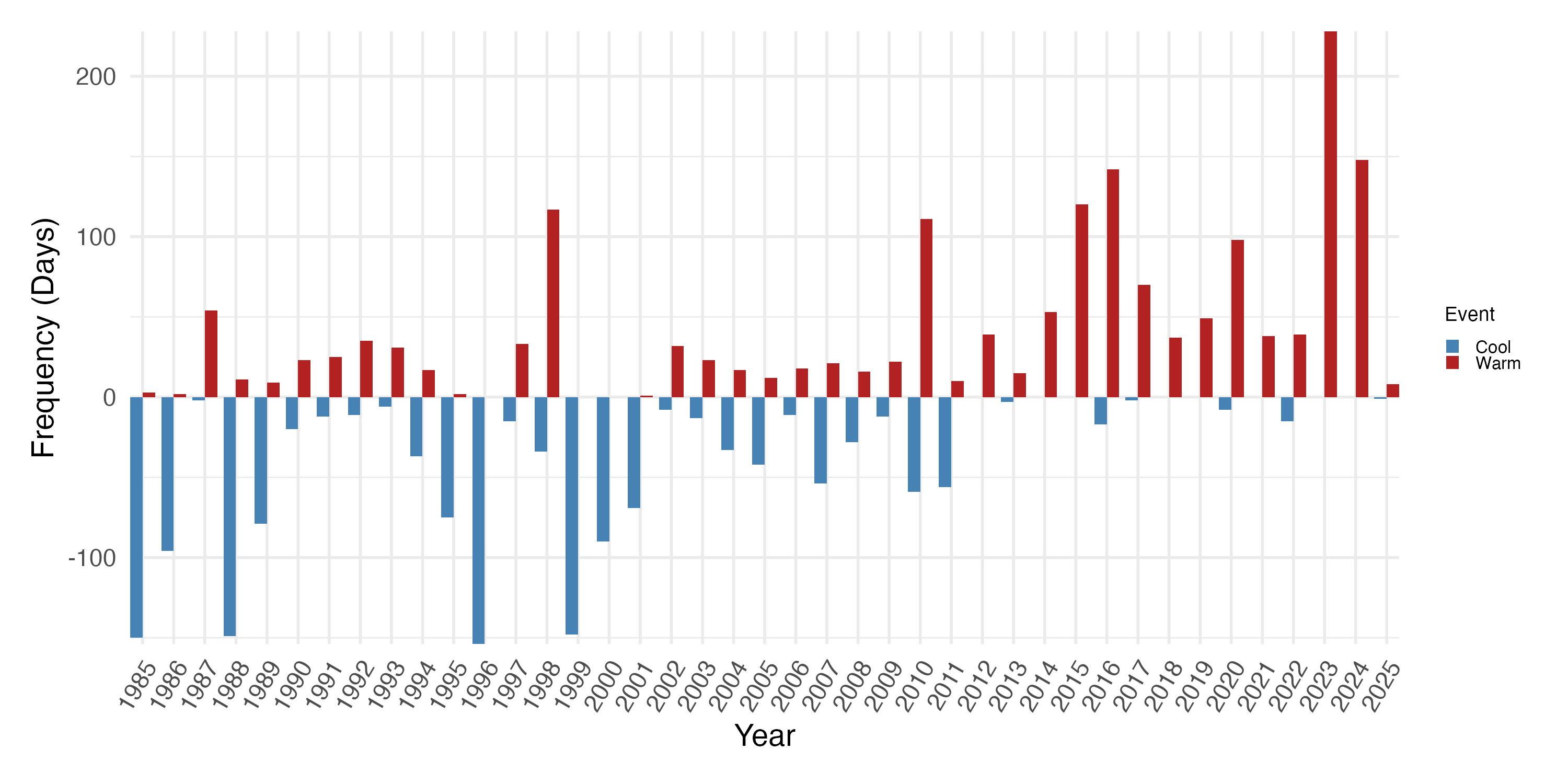

Supplement: Supplemental Information 1 — Warm events are defined as days when SST exceeds the bleaching threshold (MMM + 0.5 °C). Cool events are defined as days when SST is below the monthly mean minus 0.5 °C. [file peerj-13-20088-s001.png]

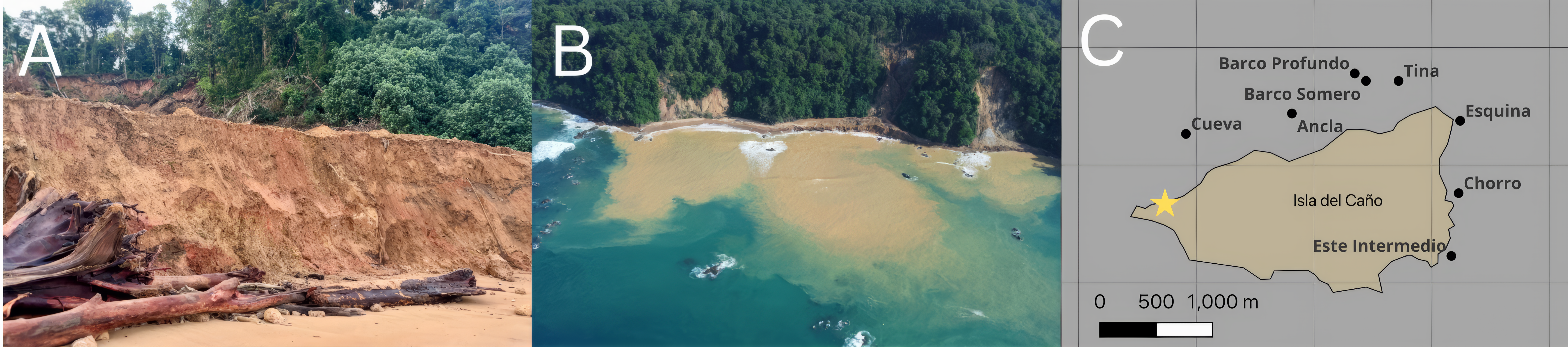

Supplement: Supplemental Information 2 — (A) the view from the beach of the extent of the landslide and amount of sediment that arrived at sea level, (B) the sediment plumes 2 months after the landslides and (C) the location of the landslides in relation to the survey sites at Isla del Caño (landslide location indicated with a star). [file peerj-13-20088-s002.png]

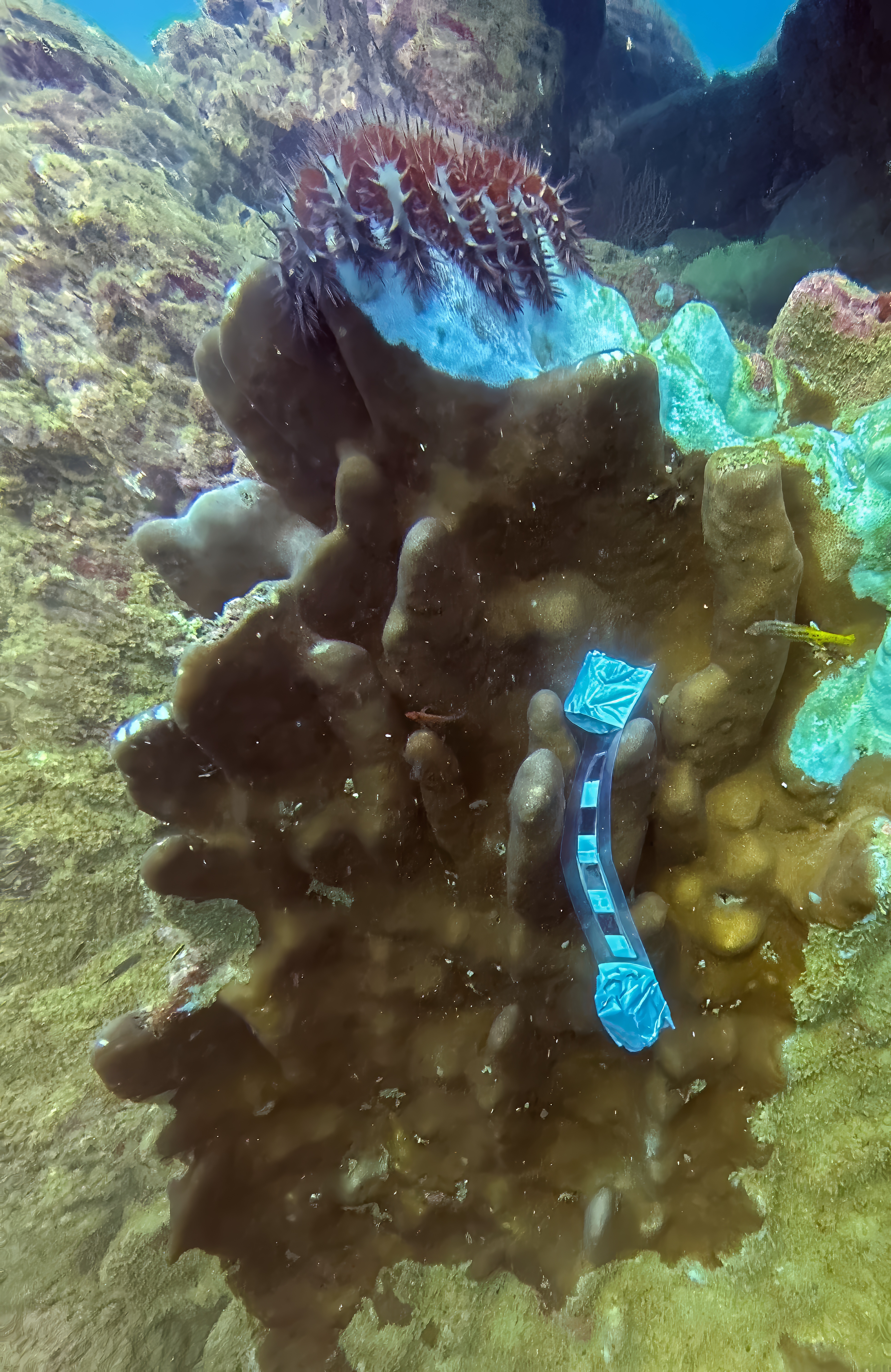

Supplement: Supplemental Information 3 [file peerj-13-20088-s003.jpg]

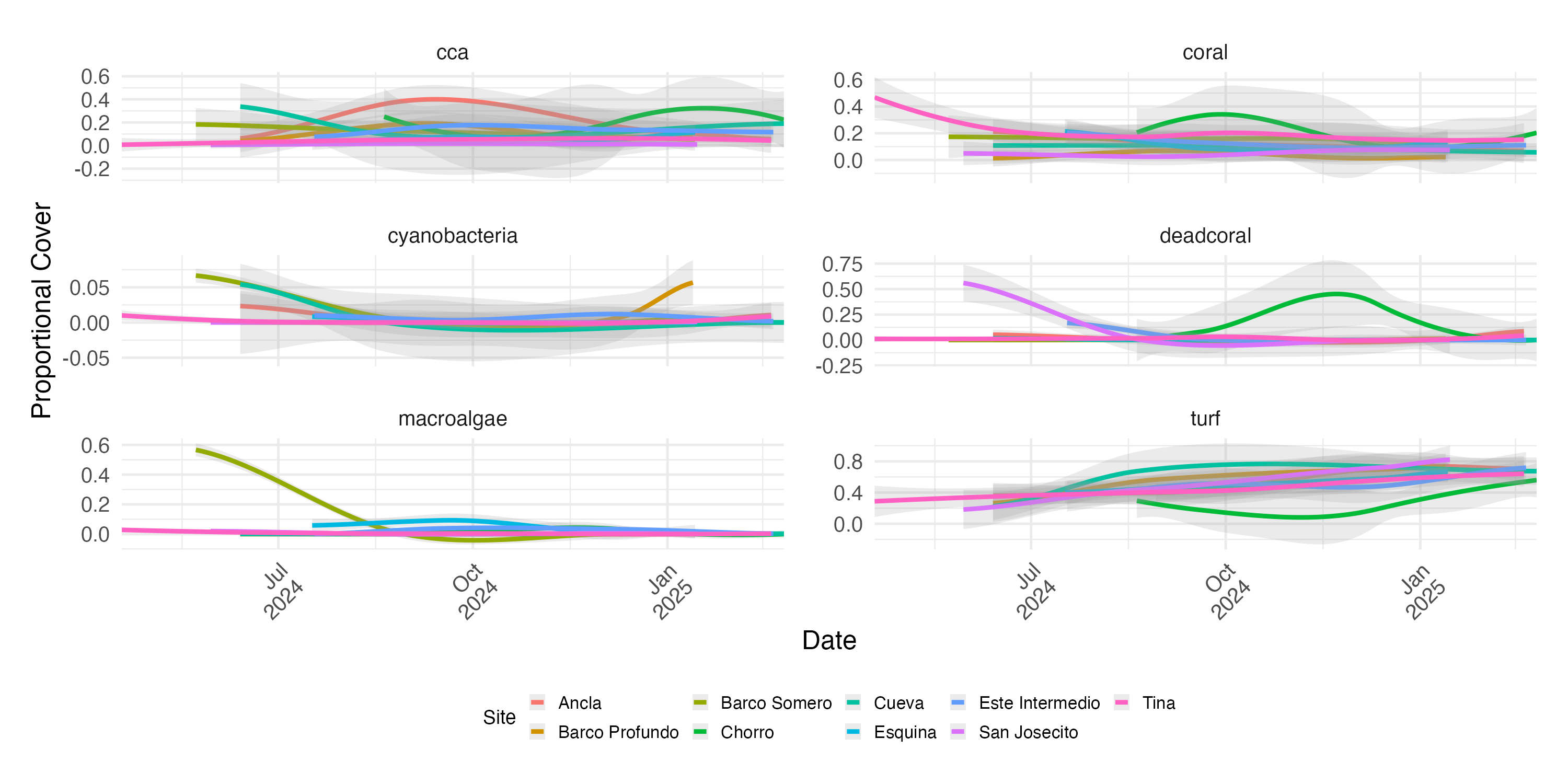

Supplement: Supplemental Information 4 — Site-level changes over time and complements the SIMPER analysis by showing the raw cover trends that contribute to compositional shifts. [file peerj-13-20088-s004.png]

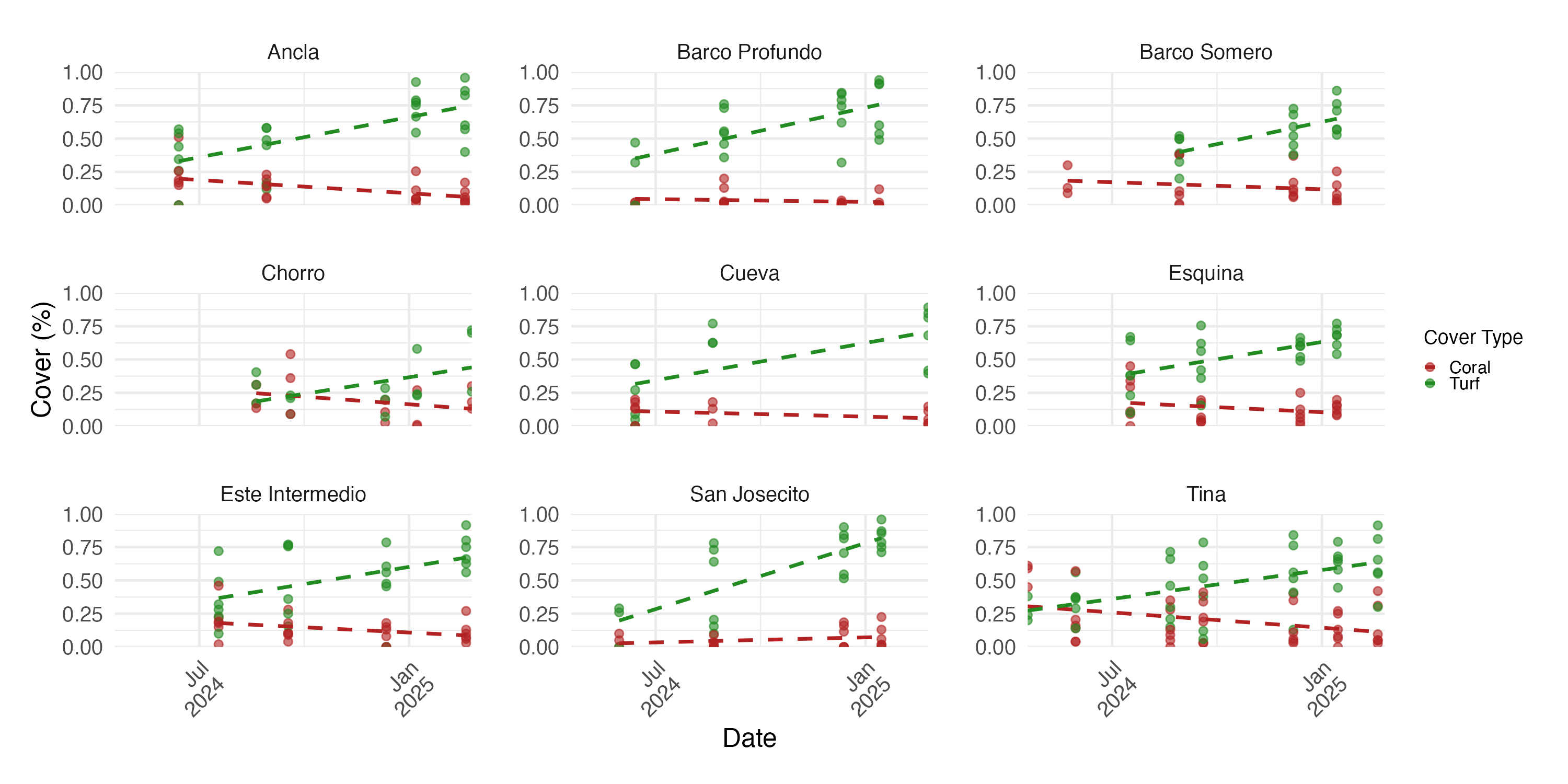

Supplement: Supplemental Information 5 [file peerj-13-20088-s005.png]

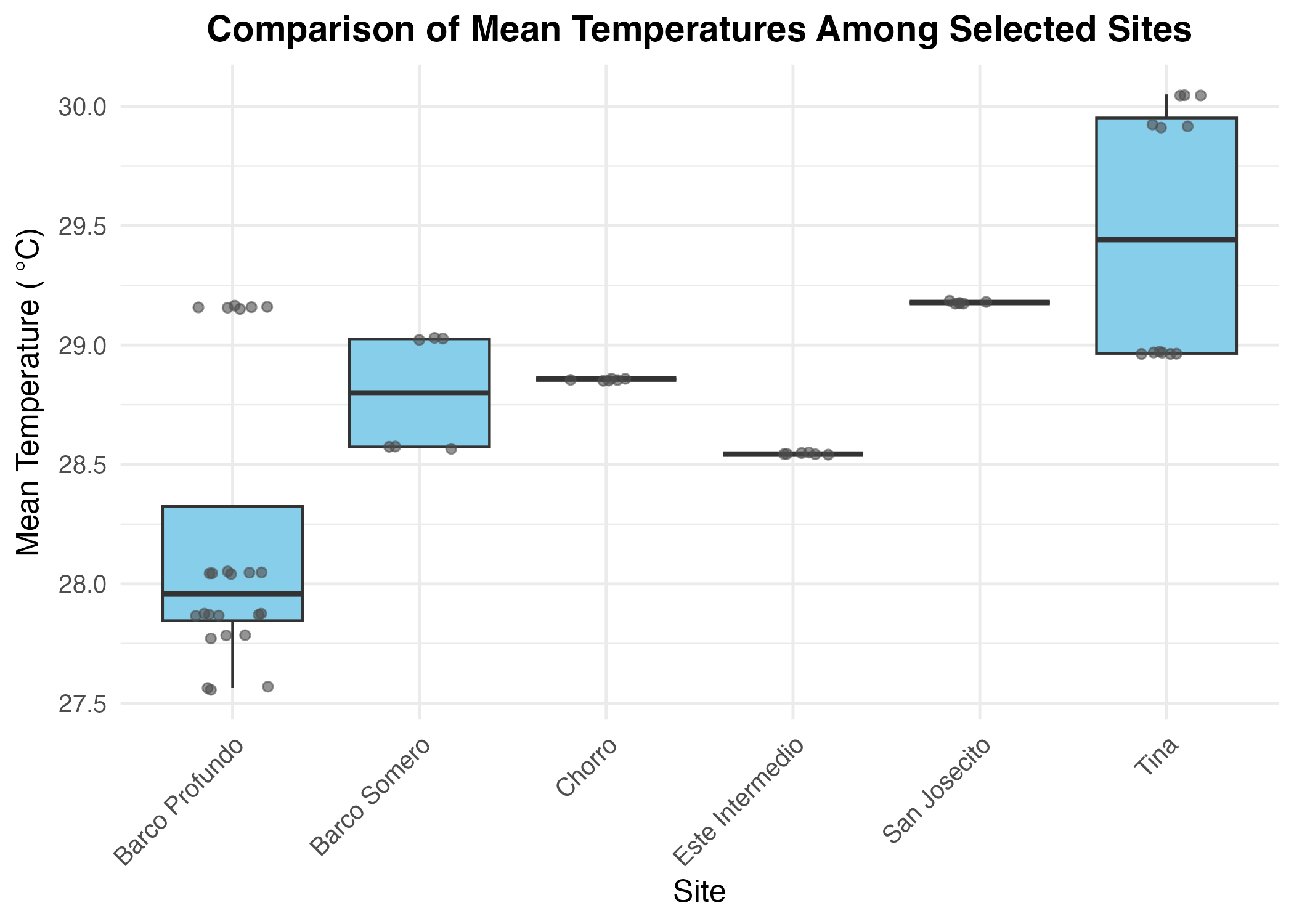

Supplement: Supplemental Information 6 [file peerj-13-20088-s006.png]

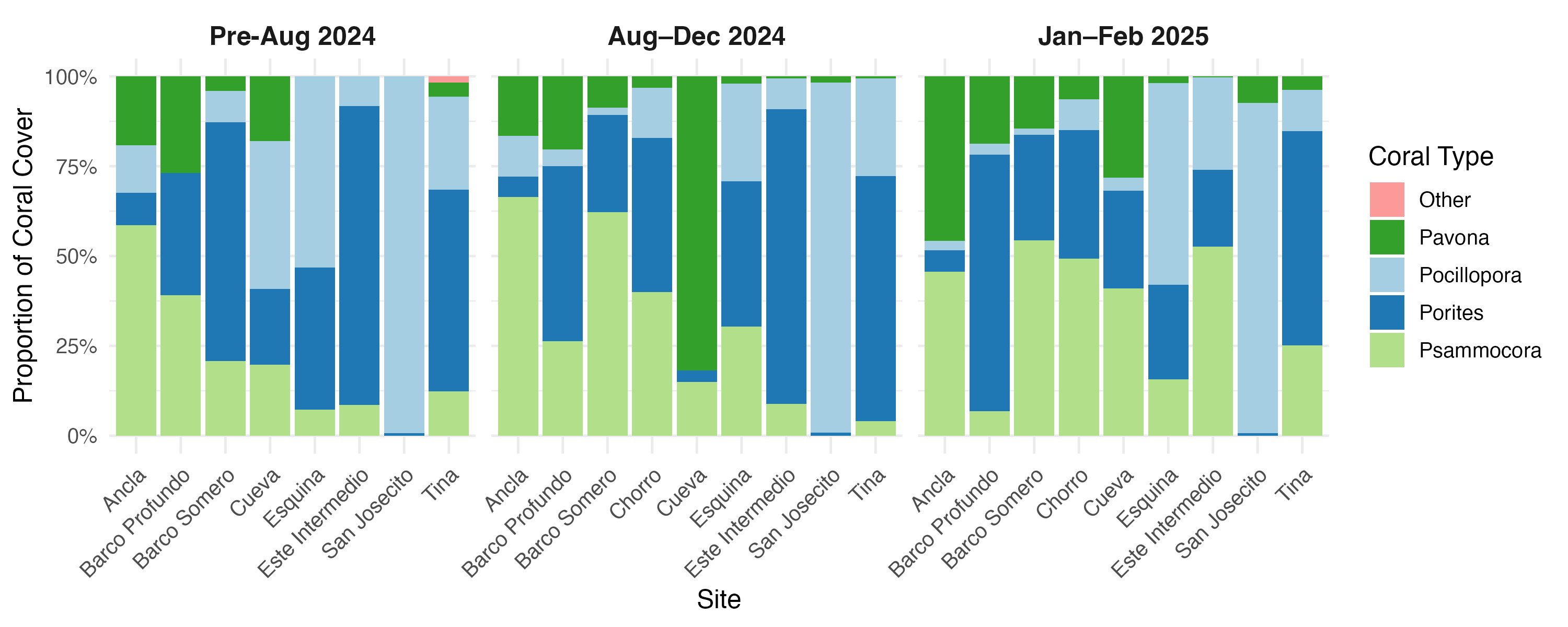

Supplement: Supplemental Information 7 [file peerj-13-20088-s007.png]

Table S5: Presence/Absence of each coral species by site at Isla del Caño and Osa Peninsula


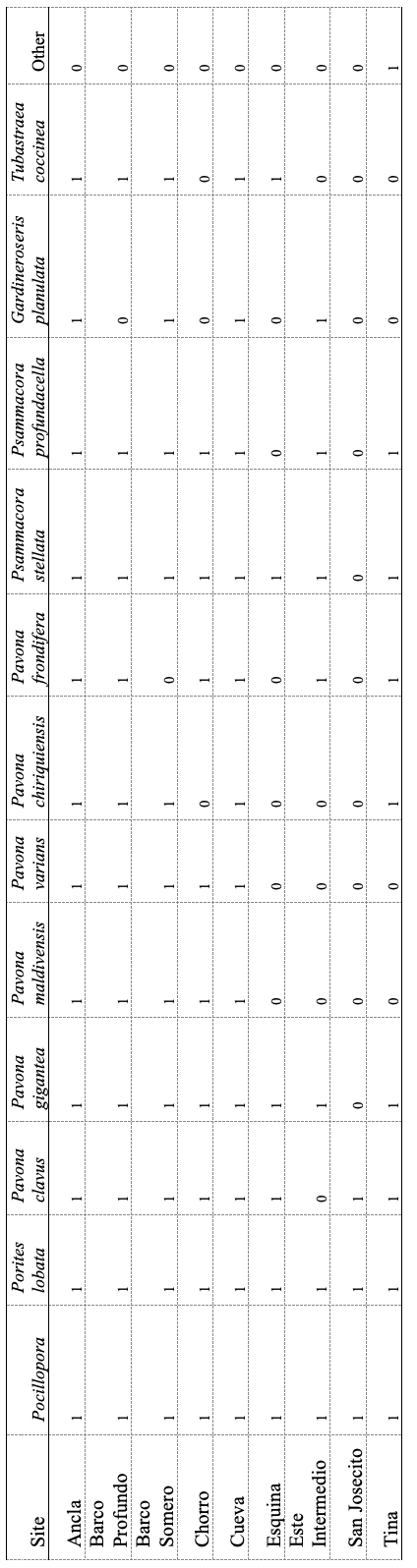

Supplement: Supplemental Information 12 [file peerj-13-20088-s012.docx]
