# Supplementary material for: El Niño-driven phase shift to algal dominance on Isla del Caño’s coral reefs: implications for urgent restoration
Source: PeerJ. 2025 Nov 20;13:e20088. doi: 10.7717/peerj.20088 (PMC12640635; doi:10.7717/peerj.20088)
Supplement: Supplemental Information 8 [file peerj-13-20088-s008.docx]

Table S1: Site coordinates and depth ranges

| **Site** | **Location** | **Min Depth (m)** | **Max Depth (m)** | **Depth Range (m)** |
| --- | --- | --- | --- | --- |
| Ancla | 8°42'51"N 83°52'54"W | 8 | 15 | 7 |
| Barco Profundo | 8°43'05"N 83°52'35"W | 13 | 16 | 3 |
| Barco Somero | 8°43'02"N 83°52'32"W | 9 | 12 | 3 |
| Chorro | 8°42'30"N 83°52'02"W | 5 | 8 | 3 |
| Cueva | 8°42'46"N 83°53'26"W | 7 | 17 | 10 |
| Esquina | 8°42'43"N 83°51'55"W | 3 | 7 | 4 |
| Este Intermedio | 8°42'07"N 83°52'01"W | 4 | 9 | 5 |
| San Josecito | 8°40'14"N 83°43'03"W | 1 | 3 | 2 |
| Tina | 8°43'01"N 83°52'22"W | 4 | 8 | 4 |
