# Supplementary material for: El Niño-driven phase shift to algal dominance on Isla del Caño’s coral reefs: implications for urgent restoration
Source: PeerJ. 2025 Nov 20;13:e20088. doi: 10.7717/peerj.20088 (PMC12640635; doi:10.7717/peerj.20088)
Supplement: Supplemental Information 9 [file peerj-13-20088-s009.docx]

Table S2: Sites and methods used for each survey since 2019-2025 in Caño Island, Costa Rica.

| **Year** | **Month** | **Sites** | **Survey Method** |
| --- | --- | --- | --- |
| 2019 | December | Chorro | 20cm point intercept 10m x 3 transects |
| 2020 | June | Chorro | 20cm point intercept 10m x 3 transects |
| 2021 | August  September | Chorro, Cueva | 20cm point intercept 10m x 3 transects |
| 2024 | February | San Josecito | 1x1m quadrat for 30m, x 3 transects |
| 2024 | March | Cueva | 1x1m quadrat for 10m, x 3 transects |
| 2024 | April | Barco Profundo, Barco Somero, Cueva, Tina, San Josecito | 1mx10m belt x 3 transects  10cm point intercept 10m x 3 transects |
| 2024 | May | Barco Somero, Chorro,  San Josecito, Tina | 1mx10m belt x 3 transects  Line intercept 10m x 3 transects |
| 2024 | June | Ancla, Cueva, Barco Profundo | 1mx10m belt x 6 transects  Line intercept 10m x 6 transects |
| 2024 | July | Esquina, Este Intermedio | 1mx10m belt x 6 transects  Line intercept 10m x 6 transects |
| 2024 | August | Ancla, Barco Profundo, Barco Somero, Chorro, Cueva, San Josecito, Tina | 1mx10m belt x 6 transects  Line intercept 10m x 6 transects |
| 2024 | September | Chorro, Este Intermedio, Esquina, Tina | 1mx10m belt x 6 transects  Line intercept 10m x 6 transects |
| 2024 | December | Tina, Barco Somero, Barco Profundo, Este Intermedio, San Josecito, Esquina | 1mx10m belt x 6 transects  Line intercept 10m x 6 transects |
| 2025 | January | Ancla, Chorro, Barco Profundo, Barco Somero, Esquina, Tina, San Josecito | 1mx10m belt x 6 transects  Line intercept 10m x 6 transects |
| 2025 | February | Ancla, Barco Somero, Tina, Este Intermedio, Cueva, Chorro | 1mx10m belt x 6 transects  Line intercept 10m x 6 transects |
