# Supplementary material for: El Niño-driven phase shift to algal dominance on Isla del Caño’s coral reefs: implications for urgent restoration
Source: PeerJ. 2025 Nov 20;13:e20088. doi: 10.7717/peerj.20088 (PMC12640635; doi:10.7717/peerj.20088)
Supplement: Supplemental Information 10 [file peerj-13-20088-s010.docx]

| Variable | x₍ᵢⱼ₎ (raw) | x̄ⱼ | sⱼ | z₍ᵢⱼ₎ = (x₍ᵢⱼ₎–x̄ⱼ)/sⱼ | Lⱼ | Dⱼ | Cⱼ = Lⱼ×Dⱼ | Contribution = Cⱼ×z₍ᵢⱼ₎ |
| --- | --- | --- | --- | --- | --- | --- | --- | --- |
| Coral cover | 45.0 | 30.0 | 10.0 | 1.50 | 2.10 | +4 | 8.40 | 12.60 |
| CCA | 5.0 | 3.0 | 1.0 | 2.00 | 1.50 | +2 | 3.00 | 6.00 |
| Turf algae | 20.0 | 25.0 | 5.0 | –1.00 | 1.80 | –3 | –5.40 | 5.40 |
| Cyanobacteria | 2.0 | 4.0 | 1.0 | –2.00 | 1.20 | –2 | –2.40 | 4.80 |
| Bleaching prevalence | 10.0 | 15.0 | 5.0 | –1.00 | 2.00 | –2 | –4.00 | 4.00 |
| Shannon diversity | 1.8 | 1.2 | 0.3 | 2.00 | 2.40 | +2 | 4.80 | 9.60 |
| **Raw ERFI** |  |  |  | **42.40** |  |  |  |  |

**Table S3. Worked example calculation of the Ecological Recovery Feasibility Index (ERFI).**

Notes:

xij: Observed value of variable j at site i.

x̄ j: Mean of variable j across all sites.

sj: Standard deviation of variable j across all sites.

zij=(xij− x̄ j)/sj: Standardized (z‐score) value.

ljk: Loading of variable j on principal component k.

Lj=∣lj1∣+∣lj2∣+∣lj3∣: Sum of absolute loadings on PCs 1–3 (PCA‐derived weight).

Dj: Expert‐assigned direction weight for variable j (positive values promote recovery; negative values inhibit recovery).

Cj=Lj×Dj: Composite weight for variable j.

**Raw ERFI** for site i: ∑j(Cj×zij).

**Normalized ERFI*** for site i:

(Raw ERFIi−min(Raw ERFI))/(max(Raw ERFI)−min(Raw ERFI))

ERFI* = (42.40 – min(ERFI)) / (max(ERFI) – min(ERFI))

For example, if min(ERFI)=10.00 and max(ERFI)=60.00, then ERFI* = (42.40–10.00)/(60.00–10.00) = 0.65.
