# Supplementary material for: El Niño-driven phase shift to algal dominance on Isla del Caño’s coral reefs: implications for urgent restoration
Source: PeerJ. 2025 Nov 20;13:e20088. doi: 10.7717/peerj.20088 (PMC12640635; doi:10.7717/peerj.20088)
Supplement: Supplemental Information 11 [file peerj-13-20088-s011.docx]

Table S4: Summary of Statistical Models Used in the Study

| **Model / Analysis** | **Response Variable** | **Model Structure** | **Predictors / Covariates** | **Justification / Notes** |
| --- | --- | --- | --- | --- |
| Coral diversity (Shannon Index) | Shannon Index (continuous) | Kruskal-Wallis + Dunn's post hoc | Site | Non-parametric tests used due to violations of normality and variance homogeneity. |
| Bleaching prevalence (site-level) | Proportion bleached (0–1) | Bayesian beta regression with random intercept | None (intercept-only), site (random effect) | Beta regression appropriate for bounded data; hierarchical structure captures spatial variation in bleaching. |
| Bleaching prevalence by genus | Proportion bleached by genus (0–1) | Multivariate zero-inflated beta (ZIB) regression | Structural zero indicators; site (random intercept) | ZIB structure accounts for excess zeros from taxon absence; multivariate format captures taxa-specific dynamics. |
| Coral cover temporal trend (global) | Coral cover (0–1) | Bayesian ZIB regression (4 models tested) | z_date (linear or polynomial), site (random intercept ± slope) | Models compared using LOO; linear model selected based on highest predictive accuracy. ZIB appropriate for zero-inflated proportional data. |
| Coral cover by site (OLS) | Coral cover (0–1) | Ordinary least squares regression (per site) | z_date | Simple linear regressions assess site-specific trends; selected for interpretability and supported by global model performance. |
| Coral cover vs depth | Coral cover (0–1) | Polynomial regression (lm(coral ~ poly(depth, 2))) | Depth (linear and quadratic terms) | Captures non-linear relationship; interpretable model of depth-driven biological thresholds. |
| Coral cover vs temperature | Coral cover (0–1) | Beta regression | Mean temperature, site | Beta model appropriate for bounded response; maximum and minimum temperature removed due to non-significance. |
| Benthic algae cover over time | Turf, CCA, macroalgae, Caulerpa, cyanobacteria (0–1) | Generalized Additive Models (GAMs) with beta distribution | z_date (smooth), site (random effect) | GAMs model non-linear trends while accounting for spatial structure; beta family suitable for proportional data. |
| Coral taxa composition over time | Proportional cover of Pocillopora, Porites, Pavona, Psammocora, Other (0–1) | Multivariate beta regression with random intercepts and slopes | z_date, site | Allows for site-specific baseline values and trends; captures heterogeneous trajectories among coral groups. |
| SIMPER analysis | Benthic composition dissimilarity | SIMPER (Bray-Curtis dissimilarity) | Time period ("during" vs "after") | Identifies key benthic groups contributing to temporal compositional change; robust to non-normal data. |
| PCA of benthic communities | Multivariate benthic variables | Principal Component Analysis (prcomp) | Coral, algae, CCA, bleached coral, etc. | Used to detect major axes of benthic community variation and ecological gradients across time periods. |
| Recovery Feasibility Index | Composite site score | Weighted PCA + ecological weighting | PCA loadings, standardized benthic variables | Integrates benthic condition and ecological importance to prioritize restoration; transparent, transferable approach. |
