# Supplementary material for: El Niño-driven phase shift to algal dominance on Isla del Caño’s coral reefs: implications for urgent restoration
Source: PeerJ. 2025 Nov 20;13:e20088. doi: 10.7717/peerj.20088 (PMC12640635; doi:10.7717/peerj.20088)
Supplement: Supplemental Information 13 [file peerj-13-20088-s013.docx]

Table S6: Shannon Index full model Dunn’s Post hoc comparison

| Comparison | Z | P.unadj | P.adj |
| --- | --- | --- | --- |
| Ancla - Barco Profundo | 3.17031398 | 0.00152274 | 0.05481875 |
| Ancla - Barco Somero | 1.58561237 | 0.11282727 | 1 |
| Barco Profundo - Barco Somero | -1.5343807 | 0.12493601 | 1 |
| Ancla - Chorro | 3.63931863 | 0.00027336 | 0.00984097 |
| Barco Profundo - Chorro | 0.04272695 | 0.96591919 | 1 |
| Barco Somero - Chorro | 1.76722203 | 0.07719105 | 1 |
| Ancla - Cueva | -0.5423707 | 0.58756315 | 1 |
| Barco Profundo - Cueva | -3.4305795 | 0.00060229 | 0.02168257 |
| Barco Somero - Cueva | -1.9792006 | 0.04779343 | 1 |
| Chorro - Cueva | -3.843188 | 0.00012145 | 0.00437207 |
| Ancla - Esquina | 3.19171469 | 0.00141431 | 0.05091515 |
| Barco Profundo - Esquina | -0.0868246 | 0.9308109 | 1 |
| Barco Somero - Esquina | 1.49787697 | 0.13416522 | 1 |
| Chorro - Esquina | -0.1429704 | 0.88631359 | 1 |
| Cueva - Esquina | 3.44887918 | 0.00056292 | 0.02026507 |
| Ancla - Este Intermedio | 5.3605849 | 8.30E-08 | 2.99E-06 |
| Barco Profundo - Este Intermedio | 2.00850305 | 0.04458986 | 1 |
| Barco Somero - Este Intermedio | 3.59320466 | 0.00032664 | 0.01175889 |
| Chorro - Este Intermedio | 2.23290792 | 0.02555502 | 0.91998062 |
| Cueva - Este Intermedio | 5.42394283 | 5.83E-08 | 2.10E-06 |
| Esquina - Este Intermedio | 2.16887022 | 0.03009254 | 1 |
| Ancla - San Josecito | 7.46783048 | 8.15E-14 | 2.94E-12 |
| Barco Profundo - San Josecito | 4.16105245 | 3.17E-05 | 0.00114042 |
| Barco Somero - San Josecito | 5.69543319 | 1.23E-08 | 4.43E-07 |
| Chorro - San Josecito | 4.63389227 | 3.59E-06 | 0.00012919 |
| Cueva - San Josecito | 7.36654106 | 1.75E-13 | 6.30E-12 |
| Esquina - San Josecito | 4.38434114 | 1.16E-05 | 0.00041881 |
| Este Intermedio - San Josecito | 2.28901345 | 0.02207857 | 0.79482856 |
| Ancla - Tina | 4.48110921 | 7.43E-06 | 0.00026732 |
| Barco Profundo - Tina | 0.79527618 | 0.42645287 | 1 |
| Barco Somero - Tina | 2.54473939 | 0.01093593 | 0.39369356 |
| Chorro - Tina | 0.88057095 | 0.37855009 | 1 |
| Cueva - Tina | 4.5917801 | 4.39E-06 | 0.00015821 |
| Esquina - Tina | 0.92969696 | 0.35252801 | 1 |
| Este Intermedio - Tina | -1.483599 | 0.13791533 | 1 |
| San Josecito - Tina | -3.9490536 | 7.85E-05 | 0.00282459 |
