# Supplementary material for: El Niño-driven phase shift to algal dominance on Isla del Caño’s coral reefs: implications for urgent restoration
Source: PeerJ. 2025 Nov 20;13:e20088. doi: 10.7717/peerj.20088 (PMC12640635; doi:10.7717/peerj.20088)
Supplement: Supplemental Information 14 [file peerj-13-20088-s014.docx]

Table S7: Mean percentage coral cover for December 2024 to February 2025 (SIMPER)

| Site | Mean_Before_% | Mean_After_% | % Change |
| --- | --- | --- | --- |
| Ancla | 21.25 | 9.78 | -53.99 |
| Barco Profundo | 4 | 3.52 | -12.11 |
| Barco Somero | 14.67 | 13.44 | -8.37 |
| Chorro | 13.17 | 16.86 | 28.07 |
| Cueva | 8.99 | 7.52 | -16.37 |
| Esquina | 21.41 | 10.59 | -50.55 |
| Este Intermedio | 20.33 | 11.11 | -45.35 |
| San Josecito | 4.17 | 5.88 | 40.91 |
| Tina | 31.17 | 16.2 | -48.02 |
