# Supplementary material for: El Niño-driven phase shift to algal dominance on Isla del Caño’s coral reefs: implications for urgent restoration
Source: PeerJ. 2025 Nov 20;13:e20088. doi: 10.7717/peerj.20088 (PMC12640635; doi:10.7717/peerj.20088)
Supplement: Supplemental Information 15 [file peerj-13-20088-s015.docx]

# Table S8. Tukey HSD Results for Site-wise Mean Monthly Temperature Comparisons

| Comparison | Difference (°C) | 95% CI Lower | 95% CI Upper | p-adjusted |
| --- | --- | --- | --- | --- |
| Barco Profundo – Ancla | -0.51 | -1.56 | 0.53 | 0.47 |
| Barco Somero – Ancla | 0.38 | -0.67 | 1.42 | 0.7 |
| Tina – Ancla | 1.12 | 0.15 | 2.09 | 0.023 |
| Barco Somero – Barco Profundo | 0.89 | -0.23 | 2.01 | 0.13 |
| Tina – Barco Profundo | 1.63 | 0.59 | 2.68 | 0.004 |
| Tina – Barco Somero | 0.74 | -0.3 | 1.79 | 0.2 |
