# Supplementary material for: El Niño-driven phase shift to algal dominance on Isla del Caño’s coral reefs: implications for urgent restoration
Source: PeerJ. 2025 Nov 20;13:e20088. doi: 10.7717/peerj.20088 (PMC12640635; doi:10.7717/peerj.20088)
Supplement: Supplemental Information 16 [file peerj-13-20088-s016.docx]

#### **Table S9. Summary of Regression Estimates for Coral Cover Trends**

| **Coral Genus** | **Mean Trend (β)** | **95% CI (Lower – Upper)** | **Site-Level Variability (SD)** | **95% CI (Lower – Upper)** |
| --- | --- | --- | --- | --- |
| **Pocillopora** | -0.48 | (-1.31, 0.37) | 0.52 | (0.02, 1.59) |
| **Porites** | 0.20 | (-0.70, 1.16) | 0.55 | (0.02, 1.70) |
| **Pavona** | 0.20 | (-0.78, 1.15) | 0.79 | (0.05, 2.04) |
| **Psammocora** | 0.24 | (-0.56, 1.03) | 0.41 | (0.01, 1.27) |
| **Other Corals** | -0.19 | (-0.84, 0.45) | 0.27 | (0.01, 0.87) |
