# Supplementary material for: El Niño-driven phase shift to algal dominance on Isla del Caño’s coral reefs: implications for urgent restoration
Source: PeerJ. 2025 Nov 20;13:e20088. doi: 10.7717/peerj.20088 (PMC12640635; doi:10.7717/peerj.20088)
Supplement: Supplemental Information 17 [file peerj-13-20088-s017.docx]

**Table S10. Mean benthic cover (% SD) at most recent survey by site**

| **Site** | **Live Coral (%)** | **Turf Algae (%)** | **Macroalgae (%)** | **Other (%)** |
| --- | --- | --- | --- | --- |
| Ancla | 5.4 ± 1.8 | 65.3 ± 9.1 | 2.2 ± 0.8 | 27.1 ± 7.0 |
| Barco Profundo | 6.1 ± 2.1 | 62.5 ± 8.7 | 2.6 ± 1.0 | 28.8 ± 6.3 |
| Barco Somero | 7.8 ± 2.5 | 55.4 ± 9.3 | 3.9 ± 1.4 | 32.9 ± 7.6 |
| Cueva | 4.9 ± 1.9 | 68.1 ± 8.5 | 3.0 ± 1.0 | 24.0 ± 6.4 |
| Chorro | 8.3 ± 2.3 | 53.8 ± 8.9 | 4.3 ± 1.2 | 33.6 ± 7.2 |
| Esquina | 5.7 ± 1.6 | 66.0 ± 8.8 | 2.8 ± 0.9 | 25.5 ± 6.1 |
| Este Intermedio | 7.1 ± 2.2 | 57.4 ± 9.0 | 3.1 ± 1.1 | 32.4 ± 7.4 |
| San Josecito | 5.2 ± 1.7 | 64.8 ± 9.5 | 2.3 ± 0.8 | 27.7 ± 6.2 |
| Tina | 9.6 ± 2.8 | 49.2 ± 7.5 | 4.2 ± 1.5 | 37.0 ± 8.0 |

Note: “Other” encompasses all remaining categories (e.g., cyanobacteria, sponges, sand)
