# Supplementary material for: El Niño-driven phase shift to algal dominance on Isla del Caño’s coral reefs: implications for urgent restoration
Source: PeerJ. 2025 Nov 20;13:e20088. doi: 10.7717/peerj.20088 (PMC12640635; doi:10.7717/peerj.20088)
Supplement: Supplemental Information 18 [file peerj-13-20088-s018.docx]

Table S11: Summary of PCA outputs

| PC | Standard deviation | Proportion of Variance | Cumulative Proportion | Time |
| --- | --- | --- | --- | --- |
| PC1 | 2.079694423 | 0.30894 | 0.30894 | Before |
| PC2 | 1.739674279 | 0.21618 | 0.52511 | Before |
| PC3 | 1.538151276 | 0.16899 | 0.69411 | Before |
| PC4 | 1.252653884 | 0.11208 | 0.80619 | Before |
| PC5 | 1.089516859 | 0.08479 | 0.89098 | Before |
| PC6 | 0.805613907 | 0.04636 | 0.93734 | Before |
| PC7 | 0.671703798 | 0.03223 | 0.96956 | Before |
| PC8 | 0.652768463 | 0.03044 | 1 | Before |
| PC9 | 2.09E-16 | 0 | 1 | Before |
| PC1 | 2.134922894 | 0.32556 | 0.32556 | After |
| PC2 | 1.839474035 | 0.24169 | 0.56725 | After |
| PC3 | 1.548285212 | 0.17123 | 0.73848 | After |
| PC4 | 1.139658358 | 0.09277 | 0.83125 | After |
| PC5 | 1.057782728 | 0.07992 | 0.91118 | After |
| PC6 | 0.884104987 | 0.05583 | 0.96701 | After |
| PC7 | 0.605157783 | 0.02616 | 0.99317 | After |
| PC8 | 0.309304658 | 0.00683 | 1 | After |
| PC9 | 2.03E-16 | 0 | 1 | After |
| PC1 | 2.035962501 | 0.34543 | 0.34543 | Latest |
| PC2 | 1.630060504 | 0.22142 | 0.56685 | Latest |
| PC3 | 1.376347643 | 0.15786 | 0.72471 | Latest |
| PC4 | 1.307900168 | 0.14255 | 0.86726 | Latest |
| PC5 | 0.849854833 | 0.06019 | 0.92745 | Latest |
| PC6 | 0.764413368 | 0.04869 | 0.97615 | Latest |
| PC7 | 0.479284636 | 0.01914 | 0.99529 | Latest |
| PC8 | 0.237758215 | 0.00471 | 1 | Latest |
| PC9 | 1.14E-16 | 0 | 1 | Latest |
