# Supplementary material for: El Niño-driven phase shift to algal dominance on Isla del Caño’s coral reefs: implications for urgent restoration
Source: PeerJ. 2025 Nov 20;13:e20088. doi: 10.7717/peerj.20088 (PMC12640635; doi:10.7717/peerj.20088)
Supplement: Supplemental Information 19 [file peerj-13-20088-s019.docx]

Table S12: Top PCA loadings summary table

| Variable | PC1 | PC2 | PC3 | PC4 | PC5 | PC6 | PC7 | PC8 | PC9 | Time |
| --- | --- | --- | --- | --- | --- | --- | --- | --- | --- | --- |
| bleached | -0.378 | -0.109 | 0.229 | 0.114 | 0.030 | 0.036 | 0.663 | 0.046 | 0.314 | Before |
| depth | 0.420 | -0.060 | 0.226 | 0.164 | 0.017 | 0.016 | 0.376 | 0.005 | 0.278 | Before |
| macroalgae | -0.048 | -0.313 | 0.089 | 0.507 | 0.149 | 0.591 | 0.067 | 0.198 | 0.359 | Before |
| turf | 0.163 | -0.405 | 0.250 | 0.172 | 0.229 | 0.188 | 0.454 | 0.189 | 0.206 | Before |
| deadcoral | -0.303 | 0.290 | 0.079 | 0.122 | 0.416 | 0.227 | 0.079 | 0.399 | 0.246 | Before |
| cca | 0.193 | -0.303 | 0.116 | 0.477 | 0.222 | 0.314 | 0.048 | 0.338 | 0.159 | Before |
| totalpoc | -0.276 | 0.363 | 0.178 | 0.261 | 0.084 | 0.179 | 0.283 | 0.236 | 0.120 | Before |
| totalpor | -0.024 | -0.486 | 0.129 | 0.081 | 0.226 | 0.345 | 0.028 | 0.468 | 0.233 | Before |
| totalpav | 0.405 | 0.234 | 0.218 | 0.009 | 0.042 | 0.030 | 0.149 | 0.025 | 0.210 | Before |
| totalpsamm | 0.351 | 0.161 | 0.289 | 0.058 | 0.044 | 0.310 | 0.030 | 0.533 | 0.430 | Before |
| totalother | 0.354 | 0.242 | 0.324 | 0.038 | 0.016 | 0.157 | 0.009 | 0.188 | 0.272 | Before |
| bleached | -0.202 | -0.356 | 0.304 | 0.128 | 0.230 | 0.316 | 0.079 | 0.123 | 0.291 | After |
| depth | 0.405 | 0.041 | 0.304 | 0.065 | 0.049 | 0.088 | 0.060 | 0.326 | 0.535 | After |
| coral | 0.030 | -0.355 | 0.230 | 0.266 | 0.514 | 0.244 | 0.076 | 0.255 | 0.130 | After |
| turf | 0.018 | 0.400 | 0.421 | 0.079 | 0.029 | 0.158 | 0.067 | 0.088 | 0.064 | After |
| cca | 0.330 | -0.235 | 0.199 | 0.177 | 0.209 | 0.158 | 0.546 | 0.148 | 0.193 | After |
| rock | -0.320 | 0.267 | 0.267 | 0.136 | 0.028 | 0.280 | 0.054 | 0.578 | 0.026 | After |
| totalpor | -0.218 | -0.379 | 0.176 | 0.295 | 0.292 | 0.140 | 0.022 | 0.054 | 0.069 | After |
| totalpav | 0.404 | 0.185 | 0.080 | 0.185 | 0.212 | 0.130 | 0.209 | 0.021 | 0.163 | After |
| totalother | 0.363 | -0.091 | 0.199 | 0.337 | 0.320 | 0.071 | 0.094 | 0.271 | 0.277 | After |
| coral | -0.427 | 0.279 | 0.015 | 0.077 | 0.095 | 0.125 | 0.199 | 0.265 | 0.529 | Latest |
| turf | 0.347 | -0.235 | 0.119 | 0.351 | 0.157 | 0.374 | 0.271 | 0.073 | 0.045 | Latest |
| depth | 0.366 | 0.304 | 0.096 | 0.120 | 0.431 | 0.054 | 0.009 | 0.603 | 0.403 | Latest |
| totalpoc | -0.148 | -0.466 | 0.347 | 0.049 | 0.028 | 0.031 | 0.628 | 0.400 | 0.065 | Latest |
| totalpor | -0.238 | 0.355 | 0.109 | 0.018 | 0.336 | 0.743 | 0.022 | 0.230 | 0.110 | Latest |
| totalpsamm | -0.325 | 0.416 | 0.119 | 0.015 | 0.138 | 0.065 | 0.454 | 0.439 | 0.472 | Latest |
| totalpav | 0.327 | 0.302 | 0.358 | 0.193 | 0.057 | 0.074 | 0.108 | 0.112 | 0.389 | Latest |
